# Modelling monetary and non-monetary flows of recreational ecosystem services in Germany

**DOI:** 10.1007/s13280-024-02081-w

**Published:** 2024-10-29

**Authors:** Johannes Hermes, Christian Albert, Christina von Haaren

**Affiliations:** Institute of Environmental Planning, Leibniz Universität Hannover, Herrenhäuser Str. 2, 30419 Hannover, Germany

**Keywords:** Cultural ecosystem services, Economic valuation, Integrating demand, supply, flow, and benefits, Mapping and assessment, National level, Recreation

## Abstract

While policy interest and research on recreational ecosystem services (RES) has increased substantially, insights into the actual flows of benefits to people have remained scarce, especially at levels beyond local communities. This paper aims to model RES Flows and Benefits for Germany in monetary and non-monetary terms. We use Germany as a case study due its diversity of landscapes and availability of relevant spatial and empirical data. We develop and apply an assessment approach that considers RES Demand and Supply based on user preferences. Our results show distinct demand–supply matches and mismatches, for example in southwest Germany, and highest flows near population centres. Monetary benefits are highest in counties with high RES Supply that are close to densely populated areas. Our results can usefully inform planning and decision-making, for example to improve and further justify destination management, landscape development, and investments in RES at local, regional, and national levels.

**Supplementary Information:**

The online version contains supplementary material available at 10.1007/s13280-024-02081-w.

## Introduction

Recreational ecosystem services (RES), understood as the contributions of nature and landscape to the recreation of people, receive significant attention in science and policy (Kadykalo et al. 2019). RES provide benefits for individuals and society through improving physical and mental health (Twohig-Bennett and Jones 2018; Lackey et al. 2021), and restoring psychophysiological and cognitive resources (Hartig 2021). High-level policies like the EU biodiversity strategy (European Commission 2013, 2020) or the UN’s System of Environmental Accounting (United Nations et al. 2021) aim to economically assess them (cf. Hein et al. 2020). The Intergovernmental Science-Policy Platform on Biodiversity and Ecosystem Services (IPBES), however, advocates for more diverse values of nature (Díaz et al. 2018; IPBES 2022). Still, their common goal is to better inform decisions ensuring a sustainable future for humanity. Environmental planning and regional governance need comprehensive information about RES qualities, quantities, and flows to help transform landscapes in response to climate change, the energy and mobility transition, or demographic changes, without compromising too much on the supply of RES.

The Common International Classification of Ecosystem Services (Haines-Young and Potschin 2010, 2018) suggests a cascade model, which was adapted to landscape planning by Haaren et al. (2014) and Albert et al. (2016a, 2016b). It states that ecosystem services (ES) are a function of the ecosystems’ capacity to provide services, the human inputs necessary to use them, and society’s demand for such services. The use or flow of ES connects the ecosystem with the socioeconomic system and leads to benefits. For RES Flow to occur, there must be a flow of people to RES Supply areas. Hence, RES Flow depends on proximity (especially regarding nearby recreation) and user movement (Costanza 2008; Syrbe and Walz 2012). This is a key difference with other ES. We follow the ES cascade defined by Albert et al. (2016b), but include different locations for demand and benefits to account for these differences (see Table S1 for definitions of the terms).

A comprehensive and spatially explicit qualitative evaluation and quantification of the whole (R)ES cascade is indispensable for supporting planning and governance (Peña et al. 2015). It could motivate and provide recommendations for landscape protection and development to increase supply or flow (Ekinci et al. 2022), or to prevent overuse (Salvatori et al. 2023). It could improve the consideration of RES in the multifunctional management of landscapes (cf. Neyret et al. 2023) and inform potential cooperation between regions or regulations on higher decision levels.

Various approaches to map and assess nature’s capacities to deliver RES exist (Hermes et al. 2018b; Cheng et al. 2019). However, the decision relevance of ES assessments is still limited because they rarely include the connection between ES supply and its beneficiaries (Longato et al. 2021; Mandle et al. 2021). Earlier studies focused on assessing ES capacities and later turned to quantifying supply and demand (Hernández-Morcillo et al. 2013; Milcu et al. 2013; Yahdjian et al. 2015). Most previous studies on RES quantification that incorporate demand and supply focus on their monetary value or the number of visits (Boerema et al. 2017; Hermes et al. 2018b). They are usually conducted on local to regional scales or cover only part of the territory. They focus on urban green space accessibility (e.g. Wüstemann et al. 2017; Weber et al. 2022), specific ecosystems or ‘touristic’ landscapes (Elsasser et al. 2016; Ghermandi 2018; Kulczyk et al. 2018; Inácio et al. 2022), or National Parks (e.g. Heagney et al. 2018; Mayer and Woltering 2018; Sinclair et al. 2020).

Most approaches consider RES Flow through visitor surveys or social media data, but fail to meaningfully connect it to the sources of recreational trips (people’s residences). Other approaches generate spatially aggregated results (e.g. Ezebilo 2016) and can give only general recommendations for planning and management. The few comprehensive large-scale studies include only areas with high RES Supply and use linear distance buffers to consider RES Flow (Paracchini et al. 2014; Vallecillo et al. 2019). Using buffers around potential destinations or sources to count potential visitors is problematic. Only the nearest sources or destinations are considered when buffers do not overlap or potential visitors are counted for multiple destinations due to overlapping buffers. Other large-scale studies incorporate supply and modelled demand to map the importance of landscapes for recreation (Schwarz-von Raumer et al. 2019; Hermes et al. 2020). Their demand model considers accessibility of potential destinations but is not yet empirically grounded. Others are based on empirical data but their approach falls short in adequately incorporating RES Supply (Sen et al. 2014). They include RES Capacities of the whole territory but ignore human inputs like recreational infrastructure or points of interest that enable or support their utilization (Sen et al. 2011). To the best of our knowledge, there are no large-scale studies analysing RES Benefit flows. Existing regional or local scale assessments (e.g. Burkhard et al. 2012; Peng et al. 2020) usually calculate them within administrative units, which is important information but does not facilitate planning responses for balancing services or for developing offsetting mechanisms between deficient and oversupplied units.

A remaining challenge is to develop comprehensive spatially-explicit indicators that adequately incorporate RES Supply, Demand, and Flow at national levels (Kulczyk et al. 2018). A particular difficulty is their dependence on user movement. Accounting for proximity or accessibility is indispensable, because RES Supply can only be enjoyed when people have access to it (Costanza 2008; Yahdjian et al. 2015). Hence, convincing models must incorporate spatial links between sources and potential destinations. Raster-based cost–distance approaches have proven useful in that regard (e.g. Ala-Hulkko et al. 2016), especially when combined with measures of attractivity of destinations (e.g. Sen et al. 2014). Their main principal is that RES-supplying areas are more likely to be visited the more accessible (in terms of travel time) and attractive (in terms of supply level) they are. Raster-based approaches require significantly less complexity and computing capacity and are, therefore, better suited for assessments at national level.

An improved assessment of RES needs to fulfil three key requirements: (1) It must combine a profound mapping of the ecosystem’s RES Supply with a convincing model of RES Demand to achieve reliable modelling of RES Flow. (2) It should replicate the trade-off between the cost of reaching a potential destination and the expected health and well-being benefits tied to its RES Supply, incorporating user preferences. (3) It needs to disentangle the complex co-dependencies between supply and demand, and fashion RES Flow indicators that can usefully inform planning and governance.

Such knowledge about demand, supply, flow, and their interrelation can support landscape planning by identifying areas where supply should be improved to meet the demand, and by motivating respective measures. Areas of high supply and flow can require measures against overuse or for protection from harmful developments (e.g. renewable energy transition (Wiehe et al. 2021) or traffic infrastructure). A monitoring would allow exploring temporal dynamics of RES (cf. Rau et al. 2020).

This paper aims to spatially model monetary and non-monetary flows of recreational ecosystem services in Germany, applying a novel method that fulfils the requirements outlined above. Our research objectives are

(i) To connect sources of NBR day trips with potential destinations through spatial modelling,
(ii) To map spatial matches and mismatches between demand at RES Supply areas and their RES Supply,
(iii) To estimate the spatial distribution of quantitative RES Flow across Germany,
(iv) To monetize the flow of RES Benefits between German counties.

We devised a raster-based spatial modelling approach consisting of four steps, each addressing one objective. All modelling operations were performed on a desktop PC using ESRI’s ArcGIS® Desktop version 10.7. We especially relied on the Spatial Analyst Toolbox.

## Materials and methods

The territory of Germany provides a valuable case study given its diversity of landscapes, harmonious spatial data, and existing results from prior national-level assessments that can serve as input data. We use data on RES Capacities across Germany from Hermes et al. (2018a), which are similar to results from Walz and Stein (2017) and Roth et al. (2018). We also use results from a national-level empirical study on the RES Demand of the German population (Hermes et al. 2021).

We focus on NBR-related day trips by car, which is the most relevant NBR trip type in Germany in terms of absolute time and cost allocation (Hermes et al. 2021). NBR day trips are defined as trips to RES-supplying areas of at least 4 h and without overnight stay. They are predominantly motivated by expected nature experiences. Including shorter and longer trips as well as different modes of transport would significantly increase complexity and could not be realized within this study.

### Preparing input data

We supplemented a pre-existing approach to map aesthetic qualities of landscapes in Germany (Hermes et al. 2018a) by incorporating human inputs like points of interest and the road and trail network suitable for recreational activities to map RES Supply levels. Those inputs turn RES Capacity into supply, support recreational use (Albert et al. 2016b; Kulczyk et al. 2018), or add value to potential destinations (Haaren et al. 2014). For a description of the modifications, see Appendix S2. We use the resulting RES Supply map as input data.

Population census points across Germany are the locations of NBR trip sources and their population in our model. We aggregated the original dataset with 1 km point spacing (inhabited 1 km^2^-cells; Federal Statistical Office (2015)) to 3 km point spacing and excluded sources with less than 300 inhabitants (0.3 inhabitants/km^2^) to reduce the required number of model iterations. The number of sources was thus reduced from 214 633 to 26 115, while still representing nearly 98% of the population. By using census points as sources, we can account for travel time through cities, which is particularly relevant in larger agglomerations.

Empirical input data stems from a nationwide representative survey on NBR preferences by Hermes et al. (2021). Their 2455 respondents reported their general behaviour regarding NBR (e.g. participation in different trip types, characteristics of trips) and their last respective trip. Results show that 56% of respondents participated in NBR day trips at least once in the year prior to the survey. The average frequency was 29 trips per person per year. Assuming an equilibrium between demand and use of ecosystems for recreational purposes, we can calculate RES Demand at sources (Burkhard et al. 2012). It is the annual number of trips emanating from each source (population × participation rate × trip frequency). Although equating demand and actual use is common practice in economics (Wolff et al. 2015), demand could be higher than use if people cannot meet their need for NBR day trips due to limited availability of RES Supply. They might choose overnight trips or other activities instead.

**Fig. 1:**
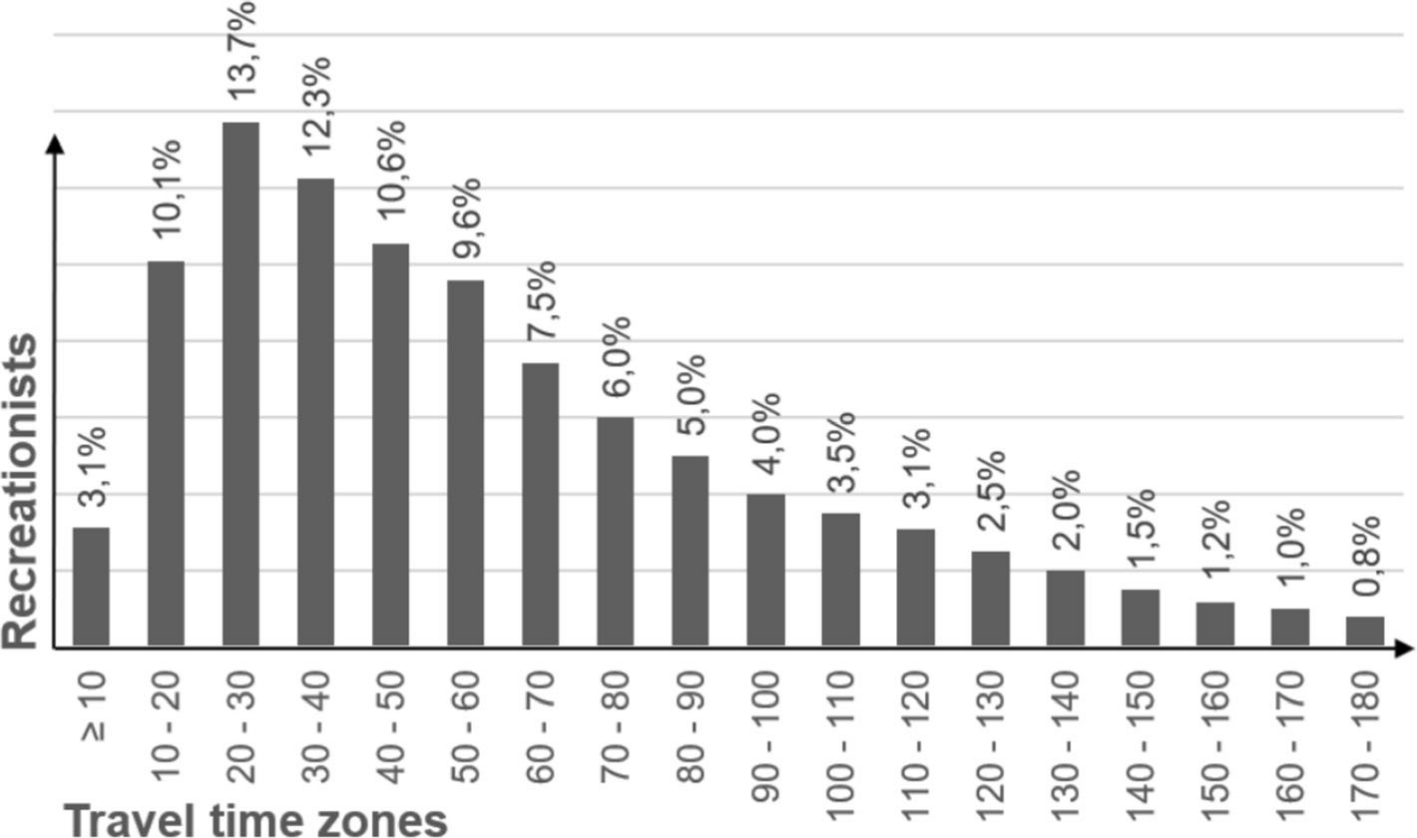
Share of surveyed day trips by car in 10-min travel time zones (one way; data: Hermes et al. (2021))

Raw data on respondents’ last trip surveyed by Hermes et al. (2021) include starting points and destinations of trips. They calculated travel time and distance for each surveyed trip using a cost–distance approach. Based on that, we assessed the share of surveyed day trips by car in 10-min increments (travel time zones, Fig. 1), which represents the travel time tolerance in our model.

We use the same cost surface as Hermes et al. (2021) to incorporate travel time in our models. It is a 1 × 1 km raster with the time needed to cross a grid cell as values. It is based on the German road network, average road speed estimates of different road types, and walking speed for cells that do not intersect roads.

We derive user preferences for RES Supply from analysing RES Supply levels at destinations of the trips surveyed by Hermes et al. (2021). More precisely, we use the density of trips per RES Supply level. First, we calculated the share of surveyed destinations per level. We extrapolated to the parent population (both from Hermes et al. 2021) to project the number of trips per level. Finally, we estimated the expected density (Table 1). We use the density because areas with high or very high RES Supply are rarer and therefore less accessible for the whole population (Bredemeier and Hermes 2019). However, if accessible, they are visited significantly more, which the trip density reflects better than the share of visits. We assume the ratios between the densities reflect the preferences and assigned respective preference factors to the RES Supply map to create the additional input for our RES Flow model.

**Table 1:** Estimated trip densities and preference factors for RES Supply levels

RES Supply level	Relative landscape quality	Area (km^2^)	Surveyed destination (%)	Projected trips [Mio.]	Trip density (Trips/km^2^)	Preference factor
Very low	0–27	28 148	4	44.07	1566	0.08
Low	27–39	73 611	19	212.08	2881	0.15
Average	39–50	120 544	36	413.15	3427	0.18
High	50–62	86 741	28	322.26	3715	0.19
Very high	62–100	19 219	13	151.49	7882	0.40
All levels		328 262		1143.05	3482	

### Spatially modelling demand at RES Supply areas

We establish a spatial link between NBR sources and RES Supply areas by distributing the RES Demand at sources within their respective accessible area. Any area (1 × 1 km grid cell) outside built-up areas can supply RES and is, therefore, a potential destination for NBR trips. Accessibility refers to the travel time needed to get from a particular source to a RES Supply area and the population’s travel time tolerance. The demand at RES Supply areas is the potential number of visits from all relevant sources per year. Our model considers the following factors:

- Locations of sources in Germany,
- RES Demand at sources,
- Travel time to relevant RES Supply areas,
- Travel time tolerance of the population, and
- Availability (amount and accessibility) of alternative destinations.

The model combines the demand indicators ‘population density’ and ‘proximity to settlement centres’ proposed by Albert et al. (2016b). In addition, it considers almost all settlements, differentiates between the locations of sources within settlements, and incorporates the availability of alternative destinations.

**Fig. 2:**
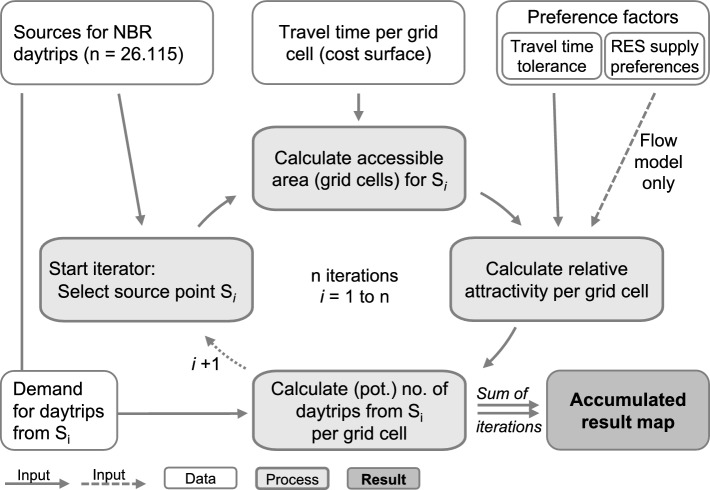
Flowchart of the model for mapping potential demand at RES Supply areas and RES Flow

Every source has limited accessible RES Supply areas, and such areas can be visited from multiple sources. We use an iterative model to account for that. It distributes the RES Demand emanating from each source separately among the relevant RES Supply areas. The accumulated number of potential visits per grid cell from all iterations then indicates the demand at RES Supply areas. The model (Fig. 2) first selects a source point. It then calculates the accessibility of relevant RES Supply areas using the cost–distance tool in ArcGIS®. Starting from the source, the tool assigns each grid cell the travel time from the source, which is accumulated over the cost surface with increasing distance. Hence, the accessible area extends further (in distance) from the source if, for example, there is a motorway nearby. This approach is more precise than Euclidean distances or distance buffers. While it is less accurate than a vector network analysis, it requires less computational power. The maximum of 70 min is a compromise between processing speed and representativeness.

Our model then calculates a relative attractivity per grid cell, depending on the required travel time from the source and the population’s travel time tolerance. We assign the respective share of trips to each cell in a travel time zone. The values of the individual cells divided by the sum of all cell values in the accessible area produce the relative attractivity of each cell. A grid cell is less attractive and, therefore, visited less frequently the more cells in similar proximity to the source and the larger the accessible area is overall. This reflects that the population may distribute thinner with more available alternatives.

The relative attractivity per grid cell (share of the demand) multiplied by the RES Demand at the source equals its potential number of visits from that source. The model adds this to results from previous iterations and continues with the next source. The accumulated number of potential visits from all iterations is the demand at RES Supply areas.

### Mapping spatial relationships between RES Demand and Supply

**Fig. 3:**
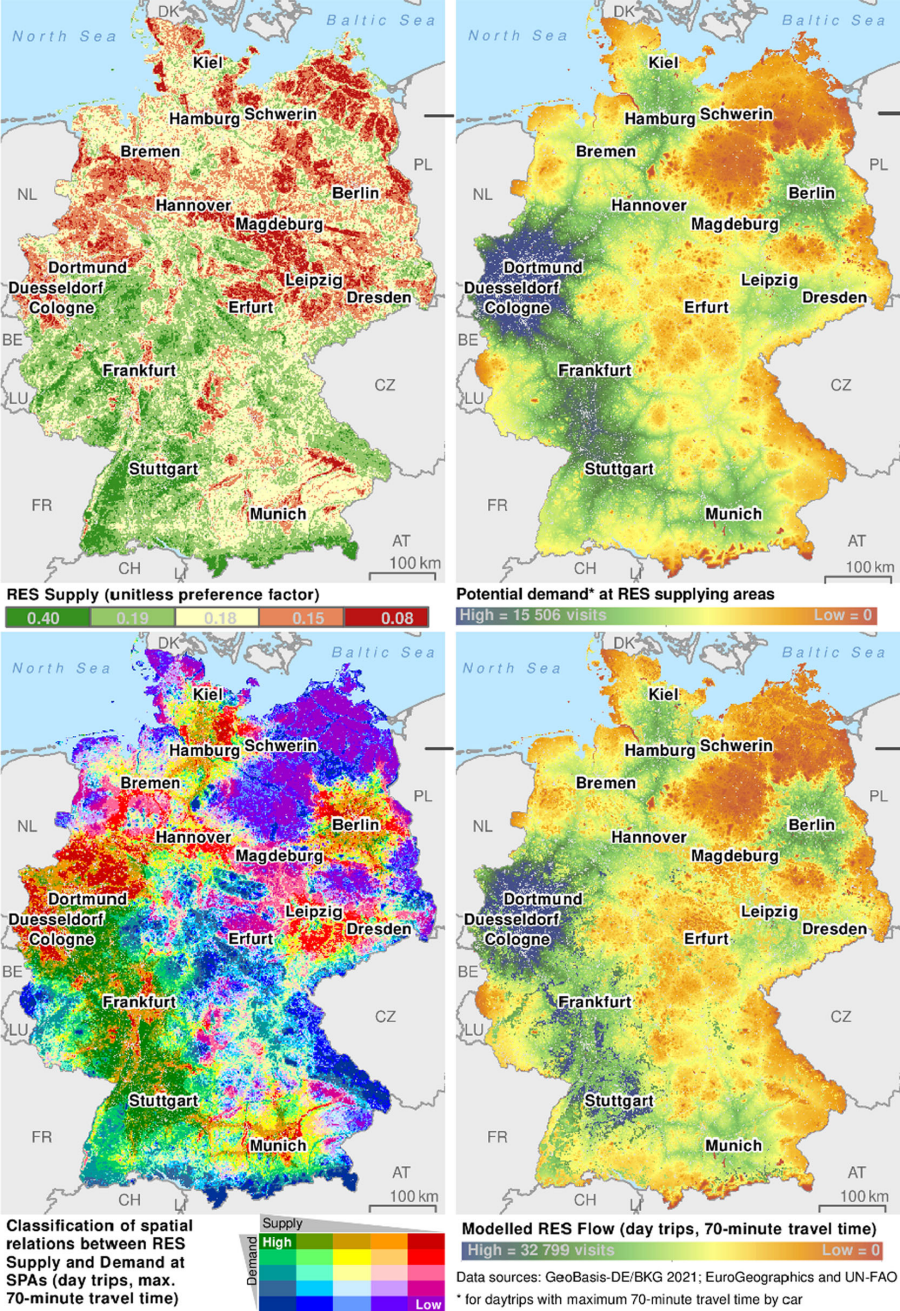
Maps of RES Supply (top left; further developed from Hermes et al. 2018a, see Appendix S2), Demand at RES Supply areas (top right), classified spatial RES Demand–Supply relationships (bottom left), and modelled RES Flow (bottom right) for Germany. Demand model specific for NBR day trips with maximum 70-min drive by car

Demand at RES Supply areas is ignorant of RES Supply levels. Therefore, some places cannot realize their potential because of low-supply and high-supply areas have to compensate for that. A spatial overlay of RES Supply and Demand there identifies spatial matches and mismatches between them. They ultimately determine the RES Flow that may be realized (Vallecillo et al. 2019). We classified the demand at RES Supply areas into quintiles, meaning that all five levels cover roughly the same area. We combined this with the RES Supply levels map, giving a unique value to every possible combination using a classification matrix (Fig. 3 bottom left). The resulting map displays the matches and mismatches between the RES Supply of a place and the RES Demand directed at it. It is a spatially explicit qualitative indicator of their relationship.

### Spatially modelling quantitative RES Flow

We quantify RES Flow to complement the descriptive information produced with the overlay matrix. The annual number of visits to a place indicates the RES Flow. It depends on its RES Supply level and respective user preferences, its accessibility from relevant sources, their RES Demand, and the supply level and accessibility of alternative destinations for the relevant sources. Low RES Demand, sparse transport infrastructure (impaired accessibility), and/or low RES Supply can restrict RES Flow. We use the same modelling approach as for the demand at RES Supply areas but include RES Supply levels and corresponding user preference factors (Table 1). Changes to the model described above (Fig. 2) only occur in step three, the calculation of relative attractivity per grid cell. We multiply the travel time tolerance factors in the accessible area with the RES Supply preference factors on a cell by cell basis before calculating the relative attractivity just like before. The RES Demand at sources is again distributed accordingly among all accessible RES Supply areas.

Attractivities per grid cell per iteration are unique to the spatial link between one source and its surrounding landscape. The resulting map, however, is a spatially explicit quantification of RES Flow that shows accumulated visits to a RES Supply area from all relevant sources.

### Monetizing the flow of RES Benefits between German counties

We distinguish individual health and well-being benefits through NBR from economic benefits. Destinations providing RES can benefit from on-site expenses on NBR trips leading to income for local businesses. This income flows from the sources to the destinations, enabling or improving the livelihood of other individuals and leading to welfare gains at societal level. As the expenses are tied to NBR trips, the income can be attributed to the RES Supply.

To calculate the benefit flow, we first multiply the RES Demand at sources and the RES Flow per grid cell with the average expenses per person per trip (EUR 15.44 (Hermes et al. 2021)). We then aggregate the resulting expected expenses (located at sources) and expected income per county using a zonal statistic (sum). The income–expense balance indicates the exchange of RES Benefits between counties. If residents realise their RES Demand inside their home county, the economic RES Benefits remain there. If they realize their demand in a neighbouring county, benefits flow (are exchanged) between them. On-site expenses represent only a fraction of the overall welfare gain, but are still relevant and relatively easy to measure and allocate. Furthermore, we can assume that the emerging patterns also reflect the health and well-being benefits that individuals derive from NBR, but with reversed polarity.

## Results

### Distribution of demand at RES Supply areas

The modelled demand at RES Supply areas accessible within a maximum 70-min drive (Fig. 3 top right) shows the highest values naturally in and around densely populated areas. By far the largest is the Rhine-Ruhr agglomeration (Cologne, Duesseldorf, Dortmund, etc.), where 10–12 million people live. The greater Berlin area, the Frankfurt/Rhine-Main area, or Munich and Stuttgart metropolitan areas have only between 5 and 6 million inhabitants. The map reflects these differences. Nationwide, the motorways make some areas accessible to people from densely populated areas. Berlin is a good illustration of how the demand at RES Supply areas decreases with increasing distance from the conurbation and of the strong influence of the road network. The example of Hamburg shows how the Elbe river represents a dividing line that leads to significantly lower values on its western side. Areas at the lower end of the scale are either sparsely populated or far away from population centres. This applies to parts of eastern Germany, the Alps in the south, and the Bavarian Forest on the border to CZ and the German coasts.

### Spatial relationships between demand and supply

The spatial classification of demand–supply relationships (Fig. 3 bottom left) reveals that landscapes between the Rhine-Ruhr area in the north and the Swabian Alb south of Stuttgart in the south are most important for NBR day trips with a maximum 70-min drive by car. High demand in these areas largely meets a high-supply level. Elsewhere, this combination only occurs on a much smaller scale south of Kiel and Berlin, between Hannover and Dortmund and north and south of Munich. Those areas are widely known in Germany as attractive destinations for NBR, which lends credibility to the modelling result.

High demand but low supply is found mainly in and around large conurbations (e.g. Hamburg, Berlin, and Munich). Expansive urban and sub-urban areas and/or intensive agriculture around them explain the low supply there.

Areas with high supply but low demand are found on the German coast and islands, in northeast Germany, between Hamburg and Hannover, in the Alps and various lower mountain ranges, and in river valleys. Those are often well-known touristic destinations. However, they are not accessible within a 70-min drive for most people.

Areas with low supply and demand are found primarily in northern and eastern Germany and, to a smaller degree, in central and southeast Germany. Those are sparsely populated agricultural landscapes.

### Distribution of RES Flow

The distribution of modelled RES Flow (bottom right) resembles the demand at RES Supply areas (i.e. the link to population density and the road network). However, a differentiation caused by the RES Supply levels is also clearly visible. For example, the RES Flow in areas with high demand but low supply is visibly lower than in neighbouring areas with similar demand but higher supply. This occurs around the Rhine-Ruhr agglomeration, where the RES Flow in some low- to medium-supply areas is indeed high in a nationwide comparison due to the higher demand. Still, neighbouring areas with higher supply generate significantly more flow.

The opposite is visible further south. Landscapes such as the Moselle and Upper Middle Rhine Valleys (between LU, Frankfurt, and Cologne) offer very high supply and are, therefore, visited more than the supply-ignorant demand at RES Supply areas suggested. The very different maxima of the two maps (RES Flow and RES Demand) results from this concentration of RES Flow in the high-supply areas.

### Flow of monetary RES Benefits between German counties

**Fig. 4:**
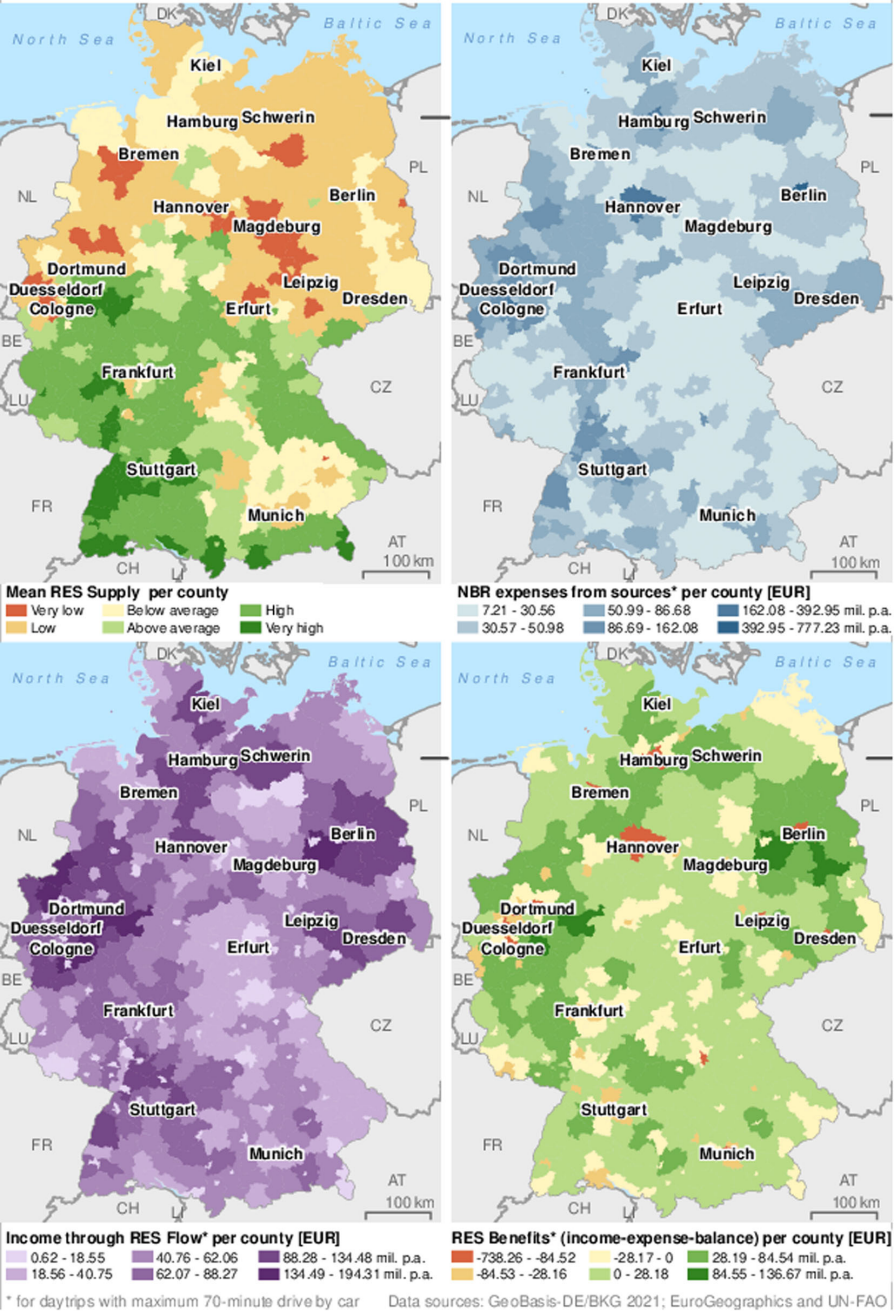
Mean RES Supply per county (top left), expected onsite expenses on NBR trips (based on Demand at sources) inside counties (top left), income from onsite expenses generated through RES Flow at RES Supply areas inside counties (bottom left), and monetary RES Benefits (income–expense balance) per county (bottom right). Results only refer to NBR day trips with maximum 70-min drive by car

The map of RES Benefits per county (Fig. 4 bottom right) shows the balance of income through realized RES Flow at indigenous RES Supply areas (Fig. 4 bottom left) and NBR expenses from indigenous sources (Fig. 4 top right). It indicates the flow or RES Benefits between counties in monetary terms. Positive values (cash inflow) show where income is higher than indigenous expenses. Conversely, negative values indicate cash outflow because indigenous expenses (and demand) are higher than the income (and flow) realized in it. Consequently, these counties receive health and well-being benefit from the RES Supply in surrounding counties.

The spatial patterns of the previous maps are present here too, but distorted due to the aggregation per county. Most counties in Germany benefit from RES-related cash inflow. However, the average is a 3.7 million EUR cash outflow. Although this is relatively close to equilibrium, it shows that the bigger part of the population cannot meet their RES Demand in their home county. Most (predominantly rural) counties have a moderately positive (0–28 million EUR) balance. They meet the demands of their population and have some visitors. This dichotomy results from the uneven distribution of population and RES Supply areas among the counties.

The RES Benefit flow between counties depends on their RES Demand at sources, their average RES Supply (Fig. 4 top left), and those of their neighbours. But their size and share of urban areas are also decisive. Berlin, Hamburg, Munich, and Cologne are urbanized to a large degree, expressed in a high population density and a big difference between average RES Supply of the landscape and of the whole territory (Table 2). They have the highest cash outflow. Hannover has a much lower population density but still the sixth-highest cash outflow. However, while more than 90% of the expenses from Berlin, Hamburg, and Munich flow to neighbouring counties, it is only 55% in Hannover. Freiburg has the ninth-highest RES Supply but still has cash outflow. A high population density, small size, and uncertainties in the model specifications described in the discussion are possible reasons. Most counties in the Rhine-Ruhr area have high to moderate cash outflow, although combined they host ~ 12% of the population. This is because their individual population densities are lower, and they provide some degree of RES Supply in close proximity. The highest cash inflows are modelled for Oberbergischer Kreis and Hochsauerlandkreis (both southeast of the Rhine-Ruhr area) due to high RES Supply and accessibility from the Rhine-Ruhr area, and for Potsdam-Mittelmark and Dahme-Spreewald, which have below national average RES Supply but benefit from the high demand in Berlin. This begs the question whether the perceived well-being benefits received in them are still similar and why this may or may not be the case. Across Germany, the modelled income attributed to RES amounts to about EUR 17.64 billion, highlighting their economic significance.

**Table 2:** RES Flow modelling results for selected counties in Germany (complete list in Table S3)

County name	Population [tsd. inh.]	Area (km^2^)	Population density [inh. /km^2^]	Mean of RES Supply areas	Mean RES Supply (whole area)	Income through RES [mil. EUR p.a.]	Expenses from sources [mil. EUR p.a.]	RES-related Benefit flow [mil. EUR p.a.]	Income–expense ratio
Berlin	3664	891	4112	37.78	18.30	38.97	777.23	− 738.42	− 95%
Hamburg	1852	755	2453	39.29	21.74	36.94	392.95	− 356.10	− 91%
Munich	1488	311	4790	34.67	14.78	11.18	315.68	− 304.57	− 96%
Cologne	1083	405	2675	37.41	22.19	48.20	229.83	− 181.68	− 79%
Hannover	1155	2297	503	35.74	30.75	110.54	245.07	− 134.58	− 55%
Freiburg	231	153	1509	64.05	49.55	7.73	48.99	− 41.26	− 84%
Hochsauerlandkreis	259	1960	132	58.34	55.07	151.5	54.95	96.54	176%
Dahme-Spreewald	173	2275	76	43.25	41.00	134.48	36.76	97.71	266%
Potsdam-Mittelmark	218	2592	84	42.08	39.98	175.11	46.23	128.87	279%
Oberbergischer Kreis	272	919	296	61.44	54.93	194.3	57.63	136.66	237%
Germany	83 155	357 580	233	45.87	42.29	~ 17 640	~ 17 640	0.00	0.00

## Discussion

We present three indicators of RES Flow, each answering different planning questions. They spatially link RES Supply to the demand directed at it and consider population density and travel time tolerance for day trips. One is a spatially explicit qualitative indicator classifying demand–supply relationships. The second is a spatially explicit quantitative indicator of RES Flow. The third is spatially aggregated. It indicates flows of monetary RES Benefit between German counties. The three indicators, along with a map of RES Supply, have different implications for planning and governance.

We model demand at RES Supply areas using the sources of NBR day trips, the road network, average road speed estimates, and travel time tolerance. While this approach is not new (e.g. Sen et al. 2014), it is still common practice, especially in larger-scale travel cost approaches, to use Euclidean distance buffers representing average travel time or cost per kilometre (Mayer and Woltering 2018; Vallecillo et al. 2019). Our results showcase the significant influence the road network has on accessibility. Furthermore, the iterative approach solves inaccuracies and limitations when considering multiple alternative destinations. Hence, we advocate using this modelling approach more frequently in travel cost approaches.^1^ However, vector network analyses are even more precise and can incorporate public transport. This is already more common on a local scale (e.g. Weber et al. 2022) and could be scaled up in future.

Our approach to mapping demand–supply relationships identifies areas of particular deficiency and need for investments in natural capital, areas with potential for development, highly important areas that might need management to avoid overuse, and areas that are less relevant for providing RES (Table 3). This is essential information for local planning authorities to develop adequate measures. Our results are more informative than the demand–supply mismatch matrix proposed by Albert et al. (2016b) or the importance matrix from Schwarz-von Raumer et al. (2019). Analysing the difference between demand at RES Supply areas and the RES Flow can also indicate potential welfare gains by investing in natural capital to increase RES Supply in high-demand areas (Albert et al. 2016b), which is crucial for informing planning and destination management. We can only provide this information by modelling demand at RES Supply areas in addition to RES Flow.

**Table 3:** Examples of how our results can inform and support planning and governance

Result	Information for planning and governance	Examples for recommendations
Demand at RES Supply areas	Accessibility of RES Supply areas Quantity of unrealized potentials (through comparison with RES Flow)	Develop areas with high potential demand Enhance access to selected areas with unrealized potentials
Spatial matches and mismatches between RES Supply and Demand	Classification of the current situation supports choice of suitable measures and prioritization	Prioritize preservation and/or management in areas with high supply and demand to prevent overuse; consider developing alternative destinations Prioritize developing RES Supply in areas with high demand but medium to low supply Prioritize preservation in areas with low demand but high supply; assess importance for tourism; consider improving day trip accessibility Prioritize other ES and land uses in areas with low demand and supply
RES Flow	Additional quantitative arguments for measures, investments, and prioritization Option for monitoring Possibility for nationwide and regional comparisons	Avoid RES-impacting projects in areas with high flow (indicates high sensitivity/potential for conflict) Prioritize other ES and RES-impacting projects in areas with low flow (consider possible touristic importance)
Flow of monetary RES Benefits	Additional monetary arguments for measures, investments, and prioritization Option for monitoring Possibility for nationwide and regional comparisons	Invest in RES Supply in counties with negative balance (this may yield welfare gains) Strengthen leisure and tourism sector, target regional marketing to neighbouring counties and further develop RES Supply in counties with positive balance Explore opportunities for mutually beneficial cooperation between neighbours with contrasting balance Use comparison results for marketing

We consider the whole territory outside built-up areas to supply a certain degree of RES. How much RES Flow is generated depends on the RES Supply, people’s preferences, and travel time tolerance. Our model reflects that even low-supply areas are used for NBR by some, if no better alternatives are available. Therefore, it is a more comprehensive evaluation of RES Flow than previous studies have presented (e.g. Sen et al. 2011, 2014; Ezebilo 2016; Mayer and Woltering 2018; Vallecillo et al. 2019). A novelty we present is the consideration of trip densities rather than visits per supply level when analysing revealed preferences. This better reflects the rarity and uneven distribution of areas of very high supply across the country (cf. Bredemeier and Hermes 2019).

Our results for the flow of RES Benefits between counties quantify a part of the return of investments in RES. They visualize the exchange of well-being and income through NBR. They identify which counties provide RES for neighbouring counties and monetize its use. This information could motivate and justify investments in RES Supply and its accessibility, and inform regional marketing. Such measures could result in more NBR-related expenses being transferred from one county inhabitant (recreationist) to another (local business owner), instead of leaving the county. And they could increase income through visitors. Furthermore, investments could reduce travel costs and CO_2_ emissions, and might increase the NBR participation rate if better NBR opportunities were available. Lastly, our results could help initiate cooperation between counties.

Monitoring our indicators could reveal changes in RES Flow over time. However, analysing the reasons for change in a particular area is difficult. It may decrease or increase for various reasons:

- Changes in RES Supply of that location,
- Changes in its accessibility (road network),
- Growing or decreasing population at relevant sources, and/or
- Changes in RES Supply and/or accessibility of alternative destinations.

In some cases, the flow could still increase while the RES Supply decreases, and vice versa. Therefore, analyses of temporal dynamics should compare changes of all three of our indicators and relate them to changes in input data (supply, population, and road network). In addition, preferences and travel time tolerance may also change over time, requiring adjustments.

RES Demand and Flow are typically measured or indicated by counting or modelling visits (demand–supply equilibrium, Wolff et al. 2015). The more visits, the higher the RES Flow. Accurate estimates of visitor numbers are more relevant than accurate estimates of the value per visit (Schägner et al. 2018). However, this approach is unable to account for demands which are not met. They refer to individual needs for recreation opportunities to reach a level of personal well-being and quality of life (Costanza et al. 2007). It also disregards that areas with high RES Supply may provide bigger individual benefits than a medium RES Supply area with the same number of visits (i.e. equal flow). Future research should focus more on this aspect.

We highlight general trends in Germany and provide initial insight into how to read and utilize them in planning and governance. Developing concrete measures requires deeper analyses of our results in local to regional case studies, supplemented with local level data. They could use the Driving forces, Pressures, State, Impacts, and Responses (DPSIR) model (Smeets and Weterings 1999), or the ‘ES-in-Planning’ framework (Albert et al. 2016a) that integrates the ES and DPSIR frameworks.

### Caveats and suggestions for improvement

A certain degree of uncertainty in the results due to model specifications was inevitable. The fixed 70-min accessible area means every grid cell in it will receive visits even if the supply is deficient and despite ample high-supply alternatives. While all landscapes are used for NBR in some capacity, this may include overestimations. This effect could be mitigated by adjusting the travel time tolerance to the local availability of RES Supply. People living in high-supply regions may be less tolerant of travel time than those in medium-supply regions. Or tolerance may increase if the available supply is low, or to reach areas with exceptional supply. Our model could be adapted to be more context sensitive, but the empirical data did not support such differentiation. The same goes for including differences between socioeconomic groups.

A test modelling of RES Flow with a maximum travel time of 180 min generated a noticeably different result (Figure S4). The overall patterns are similar, but some areas that were now accessible to more people gained in RES Flow, while some medium- and low-supply areas lost. Overall, the RES Flow is distributed wider over German landscapes, as indicated by a much lower maximum. It shows the high influence of assumptions on modelling results. It also stresses that our results are only applicable for day trips.

Further developing the flow model to incorporate accumulated travel costs from various sources to a RES Supply area to express appreciation in monetary terms would be possible. This would follow the latest recommendations for economic valuation and environmental accounting by the United Nations et al. (2021).

Lastly, a larger sample would allow for a more advanced statistical analysis of the correlations between RES Supply and travel time tolerance. It would also enable incorporating socio-demographic and regional differences, and modelling RES Flow on shorter leisure activities and multi-day trips. The modelling approach we present is suitable for integrating such results.

## Conclusions

This study contributes to the state of knowledge in mapping and assessing recreational ecosystem services by evaluating the whole RES cascade from supply to benefits with a consistent approach. Especially the nationwide modelling of RES Flow from all (semi-)natural areas linking RES-supplying and demanding areas and their respective attributes is an important step towards better considering RES in planning and governance. Our results can inform local planners and decision makers about where measures are necessary or desirable and what kind of measures (development, protection, and management) would be adequate. This aids the efficient use of funds to improve RES. Furthermore, monetizing the flow of RES Benefits between counties can motivate action and steer investments in natural capital by making potential benefits and their flow more tangible. This could facilitate cooperation between RES-supplying regions and those demanding it. Our results also support a better consideration of spatial demand–supply relations in spatial planning and impact assessments, e.g. when allocating impacting development projects (e.g. renewable energy transition (Wiehe et al. 2021) or traffic infrastructure).

The maps produced for Germany highlight the importance of RES Supply in close proximity to urban agglomerations like the Rhine-Ruhr area or Berlin. While this is no surprise, we can now express the demand, flow, and associated benefits in quantitative and monetary terms. The consistent approach means that results are comparable between regions. They show, for example, that a very high demand meets a very high supply only in southwest Germany. Still, cash inflow for counties there is on a similar level as for counties providing RES Supply for Berlin, even though the average supply level there is much lower.

Monitoring RES Supply, Demand, and Flow using our approach can provide valuable insights into their spatio-temporal dynamics. For this purpose, we recommend applying our models at regular intervals with updated spatial data. Since preferences change much slower than land use and infrastructure, a survey could be repeated at longer intervals.

Taken together, we showed that modelling RES Flow can answer diverse questions around safeguarding and improving RES. Further research should focus on a deeper dissection of our results, on integrating modelling results for shorter leisure activities and multi-day trips, and on empirical validation in selected case studies.

## Supplementary Information

Below is the link to the electronic supplementary material.

Supplementary file1 (PDF 1218 KB)

## Footnotes

1 Data are available at https://doi.org/10.25835/0006102.
