# Supplementary material for: Modelling monetary and non-monetary flows of recreational ecosystem services in Germany
Source: Ambio. 2024 Oct 29;54(2):270–84. doi: 10.1007/s13280-024-02081-w (PMC11662117; doi:10.1007/s13280-024-02081-w)

Ambio
Supplementary Information
This supplementary information has not been peer reviewed.

Title: Modelling flows of recreational ecosystem services in Germany

Content

Table S1: Terms and definitions for the RES cascade 2
Appendix S2: Adjustment and further development of the assessment of the aesthetic quality of landscapes in Germany by Hermes et al. (2018). 3
Modifications to Hermes et al. (2018) 3
Additions to better represent RES Supply 4
Result: Supply of recreational ecosystem services in Germany 4
REFERENCES 5
Table S3: RES Flow modelling results for German counties 6
Figure S4: Modelled RES Flow in Germany considering maximum 180-minute travel time 23

Table S1: Terms and definitions for the RES cascade

Term	Definition
Recreational Ecosystem Services (RES)	RES are biotic and abiotic characteristics of nature that enable activities promoting health, recuperation or enjoyment through active or immersive and/or passive or observational interactions (Haines-Young and Potschin 2018). These are direct, in-situ and outdoor interactions that depend on presence in an environmental setting. RES and additional ecosystem services form the broader category of Cultural Ecosystem Services (CES, Haines-Young and Potschin 2018).
Nature-Based Recreation (NBR)	NBR is the term for all recreational activities that involve some elements of nature, for example, terrain, plant or animal species, or water features.
RES Capacity	RES Capacity is nature's contribution to generating RES. It may provide benefits to humans today or in the future. It is usually valued by humans but not necessarily used today (Haaren et al. 2014).
Human inputs	Human inputs are the anthropogenic contributions to generating RES, including the development of recreational infrastructure. They convert RES Capacity into RES Supply.
RES Supply	RES Supply is the actual provision of RES in a particular place (service-providing area), i.e. quality of opportunities for NBR. All places outside built-up areas can supply RES (of different quality) and are potential destinations for NBR trips.
Sources	Sources of NBR trips (or service-benefitting areas) are the areas (settlements) where RES demand and beneficiaries reside. Beneficiaries use RES at supplying areas, but 'carry' benefits back to the sources.
RES Demand at sources	RES Demand at sources refers to the need for NBR by the population (Albert et al. 2016). It depends on the size of the population and their willingness to interact with the ecosystem, i.e., to take NBR trips. This demand resides at sources but is directed towards the landscape, where RES Supply can be found.
Potential demand at RES supplying areas	This is the demand directed at RES supplying areas. Spatial links between demanding and supplying areas are crucial when modelling RES, because RES Flow depends on user movement and proximity (Costanza 2008). Proximity and transport network determine the permeability of the space between sources and supplying areas (Syrbe and Grunewald 2017). The RES Supply area relevant for a source is limited, because poor permeability creates socioeconomic trade-offs. Conversely, a RES Supply area is accessible form a limited number of sources. Accounting for these spatial links allows for identifying the relevant RES Supply areas for a given source and distributing its demand among them. Potential Demand at RES Supply areas is the accumulated share of demand from all relevant sources. It depends on RES Demand at relevant sources, their proximity, travel time tolerance of the population, and availability of alternative destinations.
RES Flow	RES Flow refers to RES that are generated (RES Supply) and actually used in a specific area and time (Albert et al. 2016). RES Flow depends on RES Supply, respective user preferences, and Potential Demand at RES Supply areas (converting RES Supply into RES Flow). In other words, RES Flow is the modelled or measured number of NBR visits per temporal and spatial unit. Other commonly used terms are RES use or utilisation (Haaren et al. 2014).
RES Benefits	RES Benefits for people result from RES Flow. They occur at different locations and levels. Individuals benefit from improved (mental) health and well-being through NBR. Communities benefit when their citizens are healthier, work more productively, and relieve the healthcare system. Destinations providing RES can benefit from on-site expenses on NBR trips leading to income for local businesses. RES Benefits thus flow (are exchanged) between RES supplying and demanding areas (sources).

Appendix S2: Adjustment and further development of the assessment of the aesthetic quality of landscapes in Germany by Hermes et al. (2018).

Hermes et al. (2018)[1] mapped the aesthetic quality of landscapes in Germany using landscape diversity, naturalness and uniqueness as established indicators for landscape attractiveness. They apply several landscape metrics as proxies to spatially evaluate and map each of them in a multi-criteria GIS analysis. The resulting values are indicative, showing where quality is higher or lower compared to other places on a scale between 0 and 100, where 100 is the highest value found in Germany.

We modified their approach to account for peer feedback and our own reflections on the original model and results, and to better represent RES Supply. Hermes et al. (2018) mapped the ecosystems' capacity to supply RES. They disregard human inputs that turn the capacity into RES Supply (Albert et al. 2016), or complement the supply. Human inputs are the network of roads and trails suitable for recreational activities to make landscapes accessible, and points of interest that add value to potential destinations (Haaren et al. 2014).

Modifications to Hermes et al. (2018)

First, we reduced the study area compared to Hermes et al. (2018). According to our definition of RES, the open landscape, as well as urban green spaces, were to be assessed. Therefore, larger continuous urban areas were excluded from the assessment. Our study area includes the area of Germany without urban areas (incl. industry and commerce) and road, rail and air traffic areas (except for disused areas), based on the DLM250 (BKG 2020) (minimum size for the areal representation usually ≥ 40 ha). However, the urban green spaces depicted in the LBM-DE (BKG 2011) according to CORINE land use classification remained part of the study area. The assessment excludes the marine area itself but considers its aesthetic influence on the land area. The resulting mask defines the area relevant to RES Supply in the assessment.

The calculated values of individual grid cells represent the value of the respective landscape metric in a circular neighbourhood with a 1 km radius around that cell. For landscape metrics that measure the density of landscape elements, we now use the distance-weighted density (kernel density) instead of the density (elements per area). Landscape elements in the neighbourhood of a cell thus lose significance as the distance to the cell increases. Furthermore, the model now normalizes the natural logarithm of the results of individual metrics. This ensures that the effect of a few areas with extremely high density is put into perspective. Previously, areas with low or medium density in relation to areas with extremely high density were no longer valued with sufficient differentiation after normalisation.

For the sub-indicator 'relief diversity', the relief energy ('elevation difference between highest and lowest point') in a 10 km radius around each grid cell is incorporated in addition to the density of contour lines in the 1 km neighbourhood. This highlights landscapes with particular long-distance views or spectacular horizons.

We changed the metric for the sub-indicator 'absence of noise' from a lump sum per road type on the basis of a linearly modelled sound propagation to one based on modelled noise bands (Esswein and Schwarz-v. Raumer 2004, data provided by Schwarz-v. Raumer). Their model uses sound propagation curves depending on traffic volume data and landscape structure.

We also improved the assessment of landscape uniqueness. In addition to the rarity of the landscape types, we now also consider whether a landscape is a riverine or hedgerow landscape according to Gharadjedaghi et al. (2004, updated in BfN (2014)) and whether it is part of a set of nationally significant landscapes in Germany according to Schwarzer et al. (2018b, 2018a). They include landscapes with special significance as natural landscapes, historically grown cultural landscapes, near-natural cultural landscapes with little technological influence, or other special individual landscapes,

[1] Preprint at https://doi.org/10.25835/0006102

e.g. landscapes dominated by mining or military activities. Both factors indicate a higher uniqueness. For areas to which they apply, the revised model increases the initial rating based on rareness alone. The updated sub-indicator value is the normalized mean level in the 1 km neighbourhood.

Additions to better represent RES Supply

Two new metrics indicate the human inputs that turn the RES Capacity into RES Supply. The density of points of interest, and the density of the road and trail network relevant for nature-based recreation.

We consider points of interest as a form of human input. They comprise a subset of the landscape elements included in the assessment of uniqueness by Hermes et al. (2018), but go beyond that. Next to its effect on the aesthetic quality, a point of interest is highlighted by more intense maintenance, provision of information on site or online and in brochures, as well as additional recreational infrastructure (e.g. a kiosk, restrooms, events, guided tours). Some may also enable specific recreational activities, like climbing rocks, bathing waters or protected areas (for wildlife watching). Additional data sets are: UNESCO natural heritage sites, Ramsar sites, Natura 2000 sites, biosphere reserves, national parks, nature parks, EU bathing waters, and a map of climbing rocks from the German Alpine Association.

For landscape accessibility we distinguish (minor) roads from trails (based on BKG (2013)). Roads are approved for general traffic and are therefore not free of traffic and noise, as preferred by many recreationists. Trails include all farm roads, paths, tracks and the like that are only approved for agricultural and forestry traffic, or are exclusively for bikers and hikers. Due to their higher suitability for recreation, trails are weighted double in the calculation of density.

All metrics where calculated for a 100 m grid, as suggested by Hermes et al. (2018). The updated results are available online (Hermes 2023). We aggregated them to a 1 km grid. This was necessary because the lower resolution is less resource intensive and therefore takes less time in the more advanced iterative modelling of RES Flow. Lastly, we classified the indicative landscape quality values into five RES Supply levels, according to the national average and standard deviation (Tab. 2 in the manuscript).

Result: Supply of recreational ecosystem services in Germany

The area of Germany – excluding built-up areas – is roughly 339.000 km². The majority of that (36 %) has a medium supply level (Fig. 3 top left in the manuscript). High supply (27 %) is more common than low supply (22 %). Very high (6 %) or very low (8 %) supply levels are naturally rare, due to the classification method. Very high and high supply is closely linked to high landscape diversity, especially relief diversity, and naturalness. Examples are the Alps, lower mountain ranges like the Black Forest, and some river valleys such as the Moselle. The opposite is true of vast, open agricultural landscapes in the northern half of Germany, especially between Hanover, Magdeburg, Leipzig and Erfurt, and in north-east Germany. Big cities like Berlin, Hamburg or Munich also have few high supply areas in the immediate vicinity, unlike e.g. the Rhine-Ruhr agglomeration (Cologne, Duesseldorf, Dortmund), Frankfurt, and Stuttgart. The cities named on the map are either state capitals or have more than 500,000 inhabitants.

REFERENCES

Albert, C., A. Bonn, B. Burkhard, S. Daube, K. Dietrich, B. Engels, J. Frommer, M. Götzl, et al. 2016. Towards a national set of ecosystem service indicators: Insights from Germany. *Ecological Indicators* 61: 38–48. doi: 10.1016/j.ecolind.2015.08.050

BfN. 2014. Schutzwürdige Landschaften. Retrieved 16 February, 2016, from https://www.bfn.de/0311_schutzw_landsch+M52087573ab0.html.

BKG. 2011. Digitales Landbedeckungsmodell für Deutschland.

BKG. 2013. Digitales Basis-Landschaftsmodell (AAA-Modellierung): Basis-DLM (AAA), 61 pp.

BKG. 2020. Digitales Landschaftsmodell 1:250 000 (DLM250).

Costanza, R. 2008. Ecosystem services: Multiple classification systems are needed. *Biological Conservation* 141: 350–352. doi: 10.1016/j.biocon.2007.12.020

Esswein, H., and H.-G. Schwarz-v. Raumer. 2004. Darstellung und Analyse der Landschaftszerschneidung in Bayern.: Endbericht einer Studie im Auftrag des Bayerischen Landesamts für Umweltschutz. Unveröffentlicht.

Gharadjedaghi, B., R. Heimann, K. Lenz, C. Martin, V. Pieper, A. Schulz, A. Vahabzadeh, P. Finck, et al. 2004. Verbreitung und Gefährdung schutzwürdiger Landschaften in Deutschland. *Natur und Landschaft* 79: 71–81.

Haaren, C. von, C. Albert, J. Barkmann, R.S. De Groot, J.H. Spangenberg, C. Schröter-Schlaack, and B. Hansjürgens. 2014. From explanation to application: introducing a practice-oriented ecosystem services evaluation (PRESET) model adapted to the context of landscape planning and management. *Landscape Ecology* 29: 1335–1346 (en). doi: 10.1007/s10980-014-0084-1

Haines-Young, R., and M. Potschin. 2018. Common International Classification of Ecosystem Services (CICES) V5.1: Guidance on the Application of the Revised Structure.

Hermes, J., C. Albert, and C. von Haaren. 2018. Assessing the aesthetic quality of landscapes in Germany. *Ecosystem Services* 31: 296–307. doi: 10.1016/j.ecoser.2018.02.015

Hermes, J. 2023. Dataset KOeSL-Ergebnisse-Geodaten. Hannover: LUIS (en). doi: 10.25835/0006102

Schwarzer, M., A. Mengel, W. Konold, N. Reppin, L. Mertelmeyer, M. Jansen, K.-H. Gaudry, and M. Oelke. 2018a. *Bedeutsame Landschaften in Deutschland*: *Gutachtliche Empfehlungen für eine Raumauswahl*. Band 1: Schleswig-Holstein und Hamburg, Niedersachsen und Bremen, Mecklenburg-Vorpommern, Nordrhein-Westfalen, Sachsen-Anhalt, Brandenburg und Berlin. Bonn - Bad Godesberg: Bundesamt für Naturschutz, 484 pp.

Schwarzer, M., A. Mengel, W. Konold, N. Reppin, L. Mertelmeyer, M. Jansen, K.-H. Gaudry, and M. Oelke. 2018b. *Bedeutsame Landschaften in Deutschland*: *Gutachtliche Empfehlungen für eine Raumauswahl*. Band 2: Rheinland-Pfalz, Saarland, Hessen, Thüringen, Sachsen, Baden-Württemberg, Bayern. Bonn - Bad Godesberg: Bundesamt für Naturschutz, 465 pp.

Syrbe, R.-U., and K. Grunewald. 2017. Ecosystem service supply and demand – the challenge to balance spatial mismatches. *International Journal of Biodiversity Science, Ecosystem Services & Management* 13: 148–161. doi: 10.1080/21513732.2017.1407362

Table S3: RES Flow modelling results for German counties

County name	Population [tsd.]	Area [km²]	Population Density [inh. / km²]	RES supply [0-100] at SPAs (excl. SBAs)		RES supply [0-100] incl. SBAs (= 0)		RES use at indigenous SPAs [mil visits p.a.]	Income at indigenous SPAs [mil. EUR p.a.]	Demand at indigenous SBAs [mil. visits p.a.]	Expenses from indigenous SPAs [mil. EUR p.a.]	RES use balance [mil. visits p.a.]	RES-related benefit flow	
				MEAN	StD	MEAN	StD						[mil. EUR p.a.]	ratio
Ahrweiler	130	787	166	59,19	6,81	55,78	15,28	6,18	95,42	1,79	27,68	4,39	67,73	245%
Aichach-Friedberg	135	780	173	44,35	7,02	41,44	12,91	2,92	45,03	1,86	28,65	1,06	16,39	57%
Alb-Donau-Kreis	198	1 359	146	53,08	9,43	49,99	15,44	5,38	83,08	2,72	42,05	2,66	41,03	98%
Altenburger Land	88	569	155	31,29	8,60	29,64	10,90	1,75	27,06	1,21	18,75	0,54	8,32	44%
Altenkirchen (Westerwald)	129	642	201	59,07	5,09	53,68	17,70	4,04	62,43	1,77	27,39	2,27	35,04	128%
Altmarkkreis Salzwedel	83	2 294	36	40,49	5,49	39,52	8,23	2,11	32,54	1,14	17,54	0,97	14,99	85%
Altötting	112	569	196	45,05	8,46	41,90	14,09	1,06	16,44	1,53	23,69	- 0,47	- 7,25	-31%
Alzey-Worms	131	588	222	55,04	11,28	51,39	17,51	4,22	65,08	1,80	27,73	2,42	37,35	135%
Amberg	42	50	839	52,72	4,30	39,56	23,12	0,11	1,68	0,58	8,92	- 0,47	- 7,24	-81%
Amberg-Sulzbach	103	1 256	82	52,87	4,85	50,95	10,98	2,85	43,96	1,42	21,85	1,43	22,11	101%
Ammerland	126	731	172	45,66	4,40	41,67	13,57	2,49	38,45	1,73	26,66	0,76	11,79	44%
Anhalt-Bitterfeld	157	1 454	108	33,11	11,55	30,93	13,86	2,82	43,57	2,16	33,36	0,66	10,22	31%
Ansbach	185	1 971	94	47,16	6,48	45,42	10,92	4,79	73,94	2,55	39,32	2,24	34,62	88%
Ansbach	42	100	417	48,91	4,70	42,38	17,19	0,26	3,95	0,57	8,84	- 0,32	- 4,89	-55%
Aschaffenburg	71	62	1135	48,46	6,85	35,00	22,47	0,25	3,87	0,97	15,03	- 0,72	- 11,16	-74%
Aschaffenburg	175	699	250	52,69	6,56	47,85	16,44	2,73	42,15	2,40	37,06	0,33	5,09	14%
Augsburg	296	147	2015	40,67	11,72	27,33	21,38	0,41	6,28	4,07	62,77	- 3,66	- 56,49	-90%
Augsburg	256	1 071	239	47,05	8,10	42,51	15,88	3,74	57,78	3,52	54,29	0,23	3,49	6%
Aurich	190	1 287	148	44,81	6,03	40,84	13,96	2,29	35,31	2,61	40,35	- 0,33	- 5,04	-12%
Bad Dürkheim	133	595	224	60,68	9,14	56,11	18,26	4,41	68,06	1,83	28,22	2,58	39,84	141%
Bad Kissingen	103	1 137	91	52,44	8,97	49,81	14,41	2,23	34,38	1,42	21,89	0,81	12,49	57%
Bad Kreuznach	159	864	184	60,30	7,78	56,58	16,34	5,07	78,25	2,18	33,68	2,89	44,57	132%
Bad Tölz-Wolfratshausen	128	1 111	115	58,22	6,31	56,22	12,28	3,44	53,15	1,76	27,20	1,68	25,95	95%
Baden-Baden	55	140	396	67,79	7,48	59,77	23,00	0,90	13,91	0,76	11,76	0,14	2,15	18%

Title: Modelling flows of RES from landscapes to people: Insights from Germany

County name	Population [tsd.]	Area [km²]	Population Density [inh. / km²]	RES supply [0-100] at SPAs (excl. SBAs) MEAN	RES supply [0-100] at SPAs (excl. SBAs) StD	RES supply [0-100] incl. SBAs (= 0) MEAN	RES supply [0-100] incl. SBAs (= 0) StD	RES use at indigenous SPAs [mil visits p.a.]	Income at indigenous SPAs [mil. EUR p.a.]	Demand at indigenous SBAs [mil. visits p.a.]	Expenses from indigenous SPAs [mil. EUR p.a.]	RES use balance [mil. visits p.a.]	RES-related benefit flow [mil. EUR p.a.]	RES-related benefit flow ratio
Bamberg	77	55	1404	41,78	7,00	26,06	20,98	0,11	1,65	1,05	16,27	- 0,95	- 14,61	-90%
Bamberg	147	1 168	126	49,89	7,12	47,65	12,45	3,13	48,33	2,03	31,29	1,10	17,04	54%
Barnim	187	1 480	127	44,10	8,19	40,75	14,09	6,55	101,06	2,57	39,75	3,97	61,32	154%
Bautzen	298	2 396	124	46,34	10,01	42,95	15,45	6,35	98,11	4,10	63,23	2,26	34,88	55%
Bayreuth	104	1 274	81	53,66	5,56	51,73	11,39	3,16	48,77	1,42	22,00	1,73	26,77	122%
Bayreuth	74	67	1107	54,48	4,70	37,39	25,58	0,14	2,17	1,02	15,71	- 0,88	- 13,54	-86%
Berchtesgadener Land	106	840	127	63,56	7,75	60,93	14,74	1,14	17,53	1,46	22,56	- 0,33	- 5,03	-22%
Bergstraße	271	719	377	56,19	11,90	49,80	21,06	4,93	76,06	3,72	57,50	1,20	18,56	32%
Berlin	3 664	891	4112	37,78	8,16	18,30	19,71	2,52	38,97	50,35	777,39	- 47,83	- 738,42	-95%
Bernkastel-Wittlich	113	1 168	96	59,18	9,45	56,82	14,83	3,87	59,82	1,55	23,91	2,33	35,91	150%
Biberach	202	1 409	143	51,93	6,42	48,92	13,64	4,07	62,86	2,78	42,91	1,29	19,95	46%
Bielefeld	334	259	1289	45,11	9,55	30,37	22,56	1,16	17,91	4,58	70,76	- 3,42	- 52,85	-75%
Birkenfeld	81	777	104	58,36	6,48	55,17	14,68	2,51	38,69	1,11	17,15	1,40	21,54	126%
Böblingen	393	618	636	58,19	6,03	49,79	21,20	3,58	55,29	5,40	83,36	- 1,82	- 28,07	-34%
Bochum	364	146	2502	39,90	8,29	18,18	20,64	1,06	16,36	5,01	77,32	- 3,95	- 60,97	-79%
Bodenseekreis	218	665	328	60,27	5,37	55,59	16,93	1,85	28,51	2,99	46,23	- 1,15	- 17,73	-38%
Bonn	331	141	2344	46,22	9,00	26,07	23,90	0,90	13,94	4,54	70,14	- 3,64	- 56,20	-80%
Börde	171	2 367	72	32,20	12,18	30,61	13,77	3,90	60,17	2,34	36,19	1,55	23,98	66%
Borken	372	1 421	262	36,59	7,87	33,45	12,71	8,93	137,94	5,11	78,90	3,82	59,03	75%
Bottrop	117	101	1167	40,72	6,01	29,47	18,91	0,99	15,22	1,61	24,91	- 0,63	- 9,69	-39%
Brandenburg an der Havel	72	230	314	49,36	6,80	42,58	18,13	0,95	14,67	0,99	15,28	- 0,04	- 0,61	-4%
Braunschweig	249	193	1290	34,01	8,48	22,94	17,39	0,50	7,66	3,42	52,74	- 2,92	- 45,08	-85%
Breisgau-Hochschwarzwald	265	1 378	192	59,57	12,26	56,43	17,88	4,66	71,93	3,64	56,20	1,02	15,73	28%
Bremen	567	318	1781	36,65	8,25	20,41	19,22	0,74	11,36	7,79	120,21	- 7,05	- 108,85	-91%
Bremerhaven	114	101	1120	42,73	6,54	27,76	21,06	0,14	2,17	1,56	24,09	- 1,42	- 21,93	-91%
Burgenlandkreis	178	1 414	126	34,27	10,52	32,07	13,20	3,93	60,69	2,44	37,68	1,49	23,01	61%

Ambio Supplementary Information

Title: Modelling flows of RES from landscapes to people: Insights from Germany

County name	Population [tsd.]	Area [km²]	Population Density [inh. / km²]	RES supply [0-100] at SPAs (excl. SBAs)		RES supply [0-100] incl. SBAs (= 0)		RES use at indigenous SPAs [mil visits p.a.]	Income at indigenous SPAs [mil. EUR p.a.]	Demand at indigenous SBAs [mil. visits p.a.]	Expenses from indigenous SPAs [mil. EUR p.a.]	RES use balance [mil. visits p.a.]	RES-related benefit flow	
				MEAN	StD	MEAN	StD						[mil. EUR p.a.]	ratio
Calw	160	797	201	64,76	6,50	59,94	18,12	5,66	87,41	2,20	33,98	3,46	53,43	157%
Celle	179	1 551	116	44,09	7,63	41,30	13,03	3,65	56,38	2,46	38,06	1,19	18,32	48%
Cham	128	1 527	84	56,52	5,89	54,62	11,73	1,94	29,92	1,76	27,18	0,18	2,75	10%
Chemnitz	244	221	1106	39,01	8,43	25,58	19,75	0,75	11,61	3,36	51,85	- 2,61	- 40,24	-78%
Cloppenburg	173	1 420	122	31,31	8,08	28,89	11,41	3,15	48,61	2,37	36,63	0,78	11,98	33%
Coburg	87	590	147	46,16	7,00	43,05	13,40	1,10	16,94	1,19	18,37	- 0,09	- 1,43	-8%
Coburg	41	48	846	49,49	7,53	33,89	23,83	0,09	1,44	0,56	8,67	- 0,47	- 7,23	-83%
Cochem-Zell	62	692	89	58,37	9,34	56,51	13,76	2,42	37,35	0,85	13,06	1,57	24,29	186%
Coesfeld	221	1 112	198	33,09	8,02	31,12	11,04	8,14	125,73	3,03	46,83	5,11	78,90	168%
Cottbus	99	166	596	42,73	7,93	31,99	19,77	0,19	2,87	1,36	20,94	- 1,17	- 18,07	-86%
Cuxhaven	199	2 059	97	44,97	5,88	42,32	12,03	4,12	63,55	2,73	42,18	1,38	21,37	51%
Dachau	155	579	268	43,92	4,75	41,23	11,50	2,56	39,58	2,13	32,91	0,43	6,67	20%
Dahme-Spreewald	173	2 275	76	43,25	7,72	41,00	12,19	8,71	134,48	2,38	36,77	6,33	97,71	266%
Darmstadt	159	122	1304	46,18	7,59	35,08	20,82	0,56	8,63	2,19	33,77	- 1,63	- 25,14	-74%
Darmstadt-Dieburg	298	659	452	48,10	12,17	42,34	19,35	3,87	59,75	4,09	63,16	- 0,22	- 3,42	-5%
Deggendorf	119	861	139	45,07	14,62	42,33	17,80	1,56	24,09	1,64	25,35	- 0,08	- 1,26	-5%
Delmenhorst	78	62	1241	42,01	3,76	24,11	20,97	0,17	2,62	1,06	16,44	- 0,90	- 13,82	-84%
Dessau-Roßlau	79	245	324	39,43	8,13	34,52	15,07	0,64	9,94	1,09	16,84	- 0,45	- 6,90	-41%
Diepholz	218	1 991	110	38,13	9,35	35,97	12,67	5,39	83,20	3,00	46,27	2,39	36,93	80%
Dillingen a.d. Donau	97	792	123	40,65	8,50	37,97	13,01	1,73	26,78	1,34	20,62	0,40	6,17	30%
Dingolfing-Landau	97	878	111	42,22	9,79	40,23	13,10	1,94	29,95	1,34	20,63	0,60	9,32	45%
Dithmarschen	133	1 428	93	37,33	11,64	35,14	14,30	2,80	43,28	1,83	28,27	0,97	15,01	53%
Donau-Ries	134	1 275	105	42,56	8,94	40,10	13,18	2,85	44,03	1,85	28,50	1,01	15,53	54%
Donnersbergkreis	76	645	117	51,73	9,54	49,49	14,07	3,28	50,57	1,04	16,03	2,24	34,55	216%
Dortmund	588	281	2094	38,23	8,05	21,28	19,92	2,17	33,52	8,08	124,69	- 5,90	- 91,17	-73%
Dresden	556	328	1693	41,95	8,68	27,93	21,02	0,93	14,34	7,64	118,01	- 6,71	- 103,68	-88%

County name	Population [tsd.]	Area [km^2]	Population Density [inh. / km^2]	RES supply [0-100] at SPAs (excl. SBAs)		RES supply [0-100] incl. SBAs (= 0)		RES use at indigenous SPAs [mil visits p.a.]	Income at indigenous SPAs [mil. EUR p.a.]	Demand at indigenous SBAs [mil. visits p.a.]	Expenses from indigenous SPAs [mil. EUR p.a.]	RES use balance [mil. visits p.a.]	RES-related benefit flow	
				MEAN	StD	MEAN	StD						[mil. EUR p.a.]	ratio
Duisburg	496	233	2130	41,17	7,79	23,09	21,25	1,78	27,44	6,81	105,21	- 5,04	- 77,77	-74%
Düren	265	941	282	39,76	14,65	34,39	19,24	6,59	101,72	3,64	56,25	2,94	45,46	81%
Düsseldorf	621	217	2854	40,23	10,33	23,22	21,37	1,73	26,75	8,53	131,65	- 6,79	- 104,91	-80%
Ebersberg	144	549	262	42,42	8,66	39,75	13,28	2,09	32,20	1,98	30,57	0,11	1,63	5%
Eichsfeld	99	943	105	46,25	10,76	44,63	13,56	1,88	29,07	1,37	21,10	0,52	7,97	38%
Eichstätt	133	1 214	110	42,74	7,09	40,77	11,33	3,35	51,71	1,83	28,25	1,52	23,46	83%
Eifelkreis Bitburg-Prüm	100	1 627	61	55,20	5,89	53,50	11,16	2,23	34,37	1,37	21,23	0,85	13,14	62%
Eisenach	42	104	403	48,91	15,48	42,75	21,74	0,25	3,93	0,58	8,90	- 0,32	- 4,98	-56%
Elbe-Elster	101	1 899	53	36,35	6,79	34,41	10,51	2,70	41,76	1,39	21,45	1,32	20,32	95%
Emden	50	112	444	43,11	5,60	33,73	18,47	0,19	2,92	0,69	10,58	- 0,50	- 7,66	-72%
Emmendingen	167	680	245	61,89	8,55	58,05	17,08	2,39	36,89	2,29	35,40	0,10	1,49	4%
Emsland	329	2 884	114	37,63	8,99	34,91	13,05	5,72	88,27	4,52	69,79	1,20	18,48	26%
Ennepe-Ruhr-Kreis	323	410	789	55,34	7,60	43,10	23,93	5,15	79,48	4,44	68,56	0,71	10,92	16%
Enzkreis	200	574	348	59,19	7,01	52,46	19,92	4,04	62,36	2,74	42,38	1,29	19,98	47%
Erding	139	871	160	40,10	8,25	38,34	11,51	3,28	50,71	1,91	29,47	1,38	21,24	72%
Erfurt	214	270	792	33,50	10,12	27,10	16,01	0,45	6,95	2,94	45,34	- 2,49	- 38,39	-85%
Erlangen	112	77	1460	40,07	8,74	27,73	19,88	0,14	2,23	1,54	23,84	- 1,40	- 21,61	-91%
Erlangen-Höchstadt	138	565	245	48,73	7,18	44,99	14,68	1,53	23,66	1,90	29,30	- 0,37	- 5,64	-19%
Erzgebirgskreis	332	1 828	182	52,56	6,79	46,77	17,65	5,15	79,53	4,56	70,42	0,59	9,11	13%
Essen	582	210	2769	49,48	12,40	24,39	26,22	2,06	31,73	8,00	123,57	- 5,95	- 91,84	-74%
Esslingen	534	641	832	61,74	10,00	50,51	25,47	4,28	66,08	7,33	113,22	- 3,05	- 47,14	-42%
Euskirchen	194	1 249	156	49,82	12,11	46,59	16,95	7,49	115,71	2,67	41,24	4,82	74,47	181%
Flensburg	90	57	1585	34,98	4,56	19,54	17,70	0,04	0,63	1,24	19,08	- 1,20	- 18,45	-97%
Forchheim	117	643	181	58,75	7,24	54,96	16,03	2,17	33,48	1,60	24,74	0,57	8,74	35%
Frankenthal (Pfalz)	49	44	1111	44,61	5,48	34,34	19,38	0,21	3,22	0,67	10,34	- 0,46	- 7,12	-69%
Frankfurt (Oder)	57	148	386	32,74	6,68	27,87	13,18	0,40	6,14	0,78	12,10	- 0,39	- 5,96	-49%
Frankfurt am Main	764	248	3077	35,03	9,02	22,58	18,26	0,86	13,28	10,50	162,12	- 9,64	- 148,84	-92%
Freiburg im Breisgau	231	153	1509	64,05	9,10	49,55	27,97	0,50	7,73	3,17	49,00	- 2,67	- 41,26	-84%

Title: Modelling flows of RES from landscapes to people: Insights from Germany

	Population	Area	Population Density	RES supply [0-100] at SPAs (excl. SBAs)		RES supply [0-100] incl. SBAs (= 0)		RES use at indigenous SPAs	Income at indigenous SPAs	Demand at indigenous SBAs	Expenses from indigenous SPAs	RES use balance	RES-related benefit flow	
**County name**	[tsd.]	[km^2]	[inh. / km^2]	MEAN	StD	MEAN	StD	[mil visits p.a.]	[mil. EUR p.a.]	[mil. visits p.a.]	[mil. EUR p.a.]	[mil. visits p.a.]	[mil. EUR p.a.]	ratio
Freising	180	800	225	44,43	7,61	41,67	13,02	3,35	51,78	2,48	38,26	0,88	13,52	35%
Freudenstadt	118	870	136	61,76	7,40	58,48	15,61	4,11	63,48	1,63	25,11	2,48	38,36	153%
Freyung-Grafenau	78	984	80	59,26	4,89	57,33	11,57	1,29	19,84	1,08	16,62	0,21	3,22	19%
Friesland	99	610	162	44,14	5,97	40,32	13,66	1,55	23,97	1,36	21,00	0,19	2,97	14%
Fulda	223	1 380	162	57,79	7,17	54,22	15,55	3,70	57,15	3,06	47,32	0,64	9,83	21%
Fürstenfeldbruck	219	435	503	37,65	8,99	33,23	14,77	1,65	25,46	3,01	46,41	- 1,36	- 20,95	-45%
Fürth	128	63	2024	39,14	7,45	24,85	19,76	0,11	1,67	1,76	27,20	- 1,65	- 25,53	-94%
Fürth	119	307	386	46,32	4,89	41,31	15,12	0,74	11,40	1,63	25,18	- 0,89	- 13,78	-55%
Garmisch-Partenkirchen	88	1 012	87	61,23	6,20	59,34	12,24	2,06	31,79	1,21	18,73	0,85	13,06	70%
Gelsenkirchen	259	105	2469	39,15	5,09	19,05	19,89	0,77	11,86	3,56	54,97	- 2,79	- 43,11	-78%
Gera	92	152	605	34,63	8,18	29,10	14,74	0,49	7,54	1,27	19,55	- 0,78	- 12,01	-61%
Germersheim	129	463	278	52,76	7,14	47,47	17,24	2,30	35,49	1,77	27,37	0,53	8,12	30%
Gießen	272	855	318	52,19	9,31	47,13	17,80	3,53	54,51	3,73	57,64	- 0,20	- 3,13	-5%
Gifhorn	177	1 568	113	42,07	5,96	39,75	11,22	3,50	53,96	2,44	37,60	1,06	16,36	44%
Göppingen	259	642	403	61,19	8,70	54,35	20,95	4,02	62,06	3,56	54,90	0,46	7,16	13%
Görlitz	251	2 111	119	44,91	11,33	40,28	17,36	3,28	50,70	3,44	53,16	- 0,16	- 2,46	-5%
Goslar	135	967	139	51,40	9,79	48,28	15,52	3,49	53,96	1,85	28,58	1,64	25,38	89%
Gotha	135	936	144	40,71	15,62	37,83	18,32	1,59	24,54	1,85	28,55	- 0,26	- 4,01	-14%
Göttingen	324	1 755	185	48,86	10,92	45,26	16,53	4,43	68,41	4,45	68,72	- 0,02	- 0,31	0%
Grafschaft Bentheim	138	982	140	42,97	5,67	39,97	12,24	2,21	34,16	1,89	29,26	0,32	4,91	17%
Greiz	97	846	114	42,39	9,54	39,95	13,55	2,81	43,45	1,33	20,51	1,49	22,94	112%
Groß-Gerau	276	453	609	42,42	10,86	36,48	17,84	2,20	33,89	3,79	58,52	- 1,59	- 24,63	-42%
Günzburg	127	762	167	47,40	5,61	43,60	13,96	2,53	39,12	1,75	27,02	0,78	12,10	45%
Gütersloh	365	969	376	42,17	9,15	36,96	16,32	5,02	77,43	5,01	77,40	0,00	0,03	0%
Hagen	189	160	1176	53,05	7,52	38,37	24,58	1,70	26,28	2,59	40,03	- 0,89	- 13,75	-34%
Halle (Saale)	238	135	1762	37,98	9,93	24,94	19,75	0,36	5,52	3,27	50,47	- 2,91	- 44,95	-89%
Hamburg	1 852	755	2453	39,29	9,88	21,74	20,87	2,39	36,94	25,46	393,03	- 23,06	- 356,10	-91%
Hameln-Pyrmont	149	798	186	44,20	7,70	41,08	13,55	3,04	46,88	2,04	31,52	0,99	15,35	49%

County name	Population [tsd.]	Area [km²]	Population Density [inh. / km²]	RES supply [0-100] at SPAs (excl. SBAs)		RES supply [0-100] incl. SBAs (= 0)		RES use at indigenous SPAs [mil visits p.a.]	Income at indigenous SPAs [mil. EUR p.a.]	Demand at indigenous SBAs [mil. visits p.a.]	Expenses from indigenous SPAs [mil. EUR p.a.]	RES use balance [mil. visits p.a.]	RES-related benefit flow	
				MEAN	StD	MEAN	StD						[mil. EUR p.a.]	ratio
Hamm	179	226	790	34,98	9,07	26,91	16,74	1,71	26,34	2,46	37,97	- 0,75	- 11,63	-31%
Harburg	256	1 248	205	45,71	6,70	41,60	14,56	6,00	92,65	3,52	54,32	2,48	38,33	71%
Harz	211	2 105	100	41,74	15,76	39,31	18,14	4,43	68,37	2,90	44,76	1,53	23,61	53%
Haßberge	84	956	88	46,62	6,94	44,83	11,25	2,00	30,88	1,16	17,88	0,84	13,00	73%
Havelland	165	1 727	95	37,55	9,89	35,46	12,91	5,07	78,31	2,26	34,94	2,81	43,37	124%
Heidekreis	141	1 881	75	48,30	7,36	46,24	12,12	7,30	112,78	1,94	29,89	5,37	82,89	277%
Heidelberg	159	109	1459	49,36	14,09	38,04	24,16	0,64	9,92	2,18	33,68	- 1,54	- 23,75	-71%
Heidenheim	133	627	212	50,15	6,88	46,16	15,10	1,84	28,37	1,83	28,18	0,01	0,19	1%
Heilbronn	346	1 100	315	53,86	8,47	48,89	17,56	6,16	95,05	4,76	73,49	1,40	21,56	29%
Heilbronn	126	100	1266	54,08	8,54	40,36	24,66	0,57	8,81	1,74	26,83	- 1,17	- 18,02	-67%
Heinsberg	256	628	408	34,07	9,12	28,27	15,26	4,12	63,60	3,52	54,41	0,60	9,19	17%
Helmstedt	92	676	135	36,88	9,67	34,34	13,20	1,86	28,71	1,26	19,42	0,60	9,30	48%
Herford	251	450	556	38,42	7,57	29,38	17,59	1,89	29,23	3,44	53,16	- 1,55	- 23,92	-45%
Herne	157	51	3052	39,56	6,66	15,31	19,71	0,31	4,82	2,16	33,30	- 1,84	- 28,48	-86%
Hersfeld-Rotenburg	120	1 098	110	54,54	5,37	51,56	13,44	2,46	38,04	1,65	25,52	0,81	12,52	49%
Herzogtum Lauenburg	199	1 263	158	39,67	11,97	37,10	15,15	4,92	75,97	2,74	42,25	2,18	33,72	80%
Hildburghausen	63	938	67	51,92	8,26	50,26	12,22	1,70	26,30	0,86	13,29	0,84	13,01	98%
Hildesheim	275	1 208	228	40,02	11,96	36,89	15,73	3,87	59,68	3,79	58,44	0,08	1,24	2%
Hochsauerlandkreis	259	1 960	132	58,34	8,27	55,07	15,64	9,81	151,50	3,56	54,96	6,25	96,54	176%
Hochtaunuskreis	237	482	492	57,56	8,19	50,57	20,31	2,68	41,32	3,26	50,34	- 0,58	- 9,02	-18%
Hof	45	58	779	46,60	4,98	34,13	21,07	0,14	2,12	0,62	9,58	- 0,48	- 7,46	-78%
Hof	95	893	106	49,04	5,09	46,58	11,80	2,22	34,31	1,30	20,05	0,92	14,25	71%
Hohenlohekreis	113	777	145	55,87	10,66	53,48	15,37	3,48	53,78	1,55	23,92	1,93	29,86	125%
Holzminden	70	694	101	52,11	7,01	49,46	13,34	2,08	32,04	0,96	14,90	1,11	17,15	115%
Höxter	140	1 201	116	45,65	10,51	43,28	14,40	3,28	50,65	1,92	29,65	1,36	21,00	71%
Ilm-Kreis	106	805	131	50,73	10,70	48,06	15,39	1,71	26,44	1,45	22,41	0,26	4,03	18%

County name	Population [tsd.]	Area [km²]	Population Density [inh. / km²]	RES supply [0-100] at SPAs (excl. SBAs)		RES supply [0-100] incl. SBAs (= 0)		RES use at indigenous SPAs [mil visits p.a.]	Income at indigenous SPAs [mil. EUR p.a.]	Demand at indigenous SBAs [mil. visits p.a.]	Expenses from indigenous SPAs [mil. EUR p.a.]	RES use balance [mil. visits p.a.]	RES-related benefit flow	
				MEAN	StD	MEAN	StD						[mil. EUR p.a.]	ratio
Ingolstadt	137	133	1027	44,12	9,59	32,12	21,27	0,40	6,23	1,88	29,06	- 1,48	- 22,83	-79%
Jena	111	115	965	54,02	7,30	44,47	21,64	0,42	6,42	1,52	23,49	- 1,11	- 17,08	-73%
Jerichower Land	89	1 577	57	37,72	10,54	36,22	12,70	2,68	41,33	1,23	18,97	1,45	22,36	118%
Kaiserslautern	106	640	166	53,53	7,28	49,44	15,85	2,95	45,50	1,46	22,56	1,49	22,94	102%
Kaiserslautern	100	140	713	57,12	8,08	45,13	24,34	0,78	11,97	1,37	21,14	- 0,59	- 9,18	-43%
Karlsruhe	308	173	1779	51,16	8,69	34,30	25,08	0,79	12,25	4,24	65,44	- 3,44	- 53,19	-81%
Karlsruhe	447	1 085	412	57,06	7,22	49,67	20,31	6,62	102,28	6,14	94,81	0,48	7,47	8%
Kassel	201	107	1882	46,30	9,13	26,15	23,96	0,19	2,87	2,76	42,66	- 2,58	- 39,79	-93%
Kassel	237	1 293	183	46,99	8,89	43,25	15,32	2,71	41,87	3,26	50,28	- 0,55	- 8,42	-17%
Kaufbeuren	45	40	1116	49,05	3,99	34,86	22,49	0,12	1,89	0,61	9,48	- 0,49	- 7,59	-80%
Kelheim	123	1 065	116	46,96	7,08	44,63	12,32	3,25	50,18	1,70	26,18	1,55	24,00	92%
Kempten (Allgäu)	69	63	1089	49,94	6,20	37,32	22,36	0,11	1,67	0,95	14,63	- 0,84	- 12,95	-89%
Kiel	247	119	2078	39,96	5,94	24,77	19,95	0,33	5,17	3,39	52,32	- 3,05	- 47,15	-90%
Kitzingen	92	684	134	40,58	12,01	38,17	15,09	1,59	24,54	1,26	19,45	0,33	5,08	26%
Kleve	314	1 233	254	38,24	8,83	34,31	14,31	8,43	130,22	4,31	66,53	4,12	63,69	96%
Koblenz	113	105	1077	53,91	11,56	39,68	25,75	0,52	8,09	1,56	24,06	- 1,03	- 15,97	-66%
Köln	1 083	405	2675	37,41	9,78	22,19	19,86	3,12	48,20	14,89	229,88	- 11,77	- 181,68	-79%
Konstanz	287	818	351	58,48	6,21	53,25	17,71	2,09	32,31	3,94	60,87	- 1,85	- 28,55	-47%
Krefeld	227	138	1646	34,87	6,81	20,59	17,93	1,04	16,01	3,12	48,13	- 2,08	- 32,12	-67%
Kronach	66	652	102	53,20	4,99	49,89	13,72	1,12	17,24	0,91	14,08	0,21	3,17	22%
Kulmbach	71	658	109	53,04	4,84	50,10	13,02	1,55	23,97	0,98	15,15	0,57	8,81	58%
Kusel	70	574	122	56,32	5,67	53,25	13,91	2,17	33,56	0,96	14,87	1,21	18,69	126%
Kyffhäuserkreis	74	1 038	71	39,87	11,09	38,25	13,41	1,83	28,20	1,01	15,60	0,82	12,60	81%
Lahn-Dill-Kreis	253	1 066	238	57,72	6,99	52,26	18,15	4,98	76,89	3,48	53,76	1,50	23,13	43%
Landau in der Pfalz	47	83	563	65,72	7,95	55,72	24,71	0,66	10,16	0,64	9,90	0,02	0,25	3%
Landsberg am Lech	121	804	150	45,12	9,68	42,28	14,42	2,90	44,75	1,66	25,68	1,24	19,07	74%
Landshut	73	66	1110	42,47	10,24	31,14	20,73	0,19	3,01	1,00	15,50	- 0,81	- 12,49	-81%

Title: Modelling flows of RES from landscapes to people: Insights from Germany

County name	Population [tsd.]	Area [km²]	Population Density [inh. / km²]	RES supply [0-100] at SPAs (excl. SBAs)		RES supply [0-100] incl. SBAs (= 0)		RES use at indigenous SPAs [mil visits p.a.]	Income at indigenous SPAs [mil. EUR p.a.]	Demand at indigenous SBAs [mil. visits p.a.]	Expenses from indigenous SPAs [mil. EUR p.a.]	RES use balance [mil. visits p.a.]	RES-related benefit flow	
				MEAN	StD	MEAN	StD						[mil. EUR p.a.]	ratio
Landshut	161	1 348	120	44,34	5,96	42,42	10,74	4,25	65,64	2,21	34,20	2,04	31,44	92%
Leer	171	1 086	158	44,70	7,38	40,07	15,30	2,73	42,13	2,36	36,38	0,37	5,75	16%
Leipzig	597	298	2006	36,49	11,64	23,03	19,89	0,81	12,51	8,21	126,77	- 7,40	- 114,25	-90%
Leipzig	258	1 651	156	40,20	11,05	36,22	15,95	6,01	92,72	3,55	54,82	2,45	37,90	69%
Leverkusen	164	79	2078	45,53	9,05	25,73	23,57	0,73	11,34	2,25	34,78	- 1,52	- 23,44	-67%
Lichtenfels	67	520	128	54,63	6,76	51,81	13,76	1,32	20,45	0,92	14,16	0,41	6,29	44%
Limburg-Weilburg	172	738	233	52,14	9,44	47,47	17,41	3,59	55,39	2,37	36,55	1,22	18,84	52%
Lindau (Bodensee)	82	323	254	54,56	4,53	51,13	13,94	0,63	9,77	1,13	17,42	- 0,50	- 7,64	-44%
Lippe	347	1 246	278	49,91	8,13	44,39	17,43	5,27	81,34	4,77	73,62	0,50	7,72	10%
Lörrach	229	807	284	66,62	6,04	61,98	17,94	2,72	42,07	3,14	48,55	- 0,42	- 6,49	-13%
Lübeck	216	214	1008	37,58	8,37	28,29	17,76	0,68	10,44	2,97	45,80	- 2,29	- 35,36	-77%
Lüchow-Dannenberg	49	1 227	40	45,27	5,10	44,39	8,03	0,96	14,75	0,67	10,29	0,29	4,46	43%
Ludwigsburg	545	687	794	50,90	11,50	42,60	21,54	3,63	56,11	7,49	115,62	- 3,85	- 59,51	-51%
Ludwigshafen am Rhein	173	77	2229	41,26	8,02	22,48	21,39	0,29	4,55	2,37	36,61	- 2,08	- 32,07	-88%
Ludwigslust-Parchim	212	4 767	44	41,24	8,89	39,98	11,28	6,89	106,34	2,91	44,95	3,98	61,40	137%
Lüneburg	184	1 328	139	46,53	5,26	43,78	12,10	3,90	60,20	2,53	39,09	1,37	21,11	54%
Magdeburg	236	201	1173	31,66	9,47	22,45	16,44	0,35	5,44	3,24	50,02	- 2,89	- 44,58	-89%
Main-Kinzig-Kreis	422	1 397	302	55,39	8,71	50,33	17,98	5,29	81,69	5,79	89,47	- 0,50	- 7,78	-9%
Main-Spessart	126	1 321	95	52,92	6,16	50,64	12,33	3,31	51,06	1,73	26,73	1,58	24,33	91%
Main-Tauber-Kreis	133	1 304	102	54,48	9,08	52,44	13,64	3,74	57,69	1,82	28,15	1,91	29,54	105%
Main-Taunus-Kreis	239	223	1075	48,00	11,35	37,26	22,36	1,14	17,58	3,29	50,76	- 2,15	- 33,19	-65%
Mainz	217	98	2222	50,92	8,75	32,49	25,45	0,45	6,97	2,98	46,07	- 2,53	- 39,09	-85%
Mainz-Bingen	212	605	349	63,10	8,56	57,09	20,24	5,09	78,59	2,91	44,88	2,18	33,71	75%
Mannheim	310	145	2136	39,59	8,94	24,35	20,50	0,55	8,51	4,26	65,71	- 3,70	- 57,20	-87%
Mansfeld-Südharz	134	1 449	92	38,98	13,52	36,83	15,86	3,35	51,73	1,84	28,36	1,51	23,36	82%

Title: Modelling flows of RES from landscapes to people: Insights from Germany

County name	Population [tsd.]	Area [km^2]	Population Density [inh. / km^2]	RES supply [0-100] at SPAs (excl. SBAs)		RES supply [0-100] incl. SBAs (= 0)		RES use at indigenous SPAs [mil visits p.a.]	Income at indigenous SPAs [mil. EUR p.a.]	Demand at indigenous SBAs [mil. visits p.a.]	Expenses from indigenous SPAs [mil. EUR p.a.]	RES use balance [mil. visits p.a.]	RES-related benefit flow	
				MEAN	StD	MEAN	StD						[mil. EUR p.a.]	ratio
Marburg-Biedenkopf	246	1 262	195	52,64	8,94	48,79	16,17	3,41	52,62	3,38	52,17	0,03	0,44	1%
Märkischer Kreis	409	1 061	385	55,49	6,88	48,48	19,53	10,84	167,31	5,62	86,70	5,22	80,60	93%
Märkisch-Oderland	197	2 159	91	34,28	9,17	32,10	12,19	6,52	100,63	2,71	41,84	3,81	58,79	141%
Mayen-Koblenz	215	818	263	49,65	12,80	44,79	19,12	3,86	59,57	2,95	45,57	0,91	14,00	31%
Mecklenburgische Seenplatte	258	5 496	47	38,80	11,92	37,94	13,10	3,84	59,25	3,55	54,75	0,29	4,50	8%
Meißen	240	1 455	165	34,10	9,60	31,48	12,95	4,18	64,51	3,30	51,00	0,88	13,51	26%
Memmingen	44	70	633	48,81	5,53	38,98	20,19	0,23	3,57	0,61	9,41	- 0,38	- 5,84	-62%
Merzig-Wadern	103	557	186	52,60	6,34	47,48	16,70	1,28	19,78	1,42	21,95	- 0,14	- 2,17	-10%
Mettmann	484	407	1189	47,95	8,00	34,09	22,76	4,47	69,03	6,66	102,76	- 2,18	- 33,73	-33%
Miesbach	100	866	116	57,07	7,63	54,84	13,35	3,05	47,08	1,38	21,26	1,67	25,83	122%
Miltenberg	129	716	180	53,15	6,36	49,40	14,93	2,57	39,60	1,77	27,31	0,80	12,29	45%
Minden-Lübbecke	310	1 152	269	37,75	7,78	32,78	14,68	3,99	61,67	4,26	65,83	- 0,27	- 4,16	-6%
Mittelsachsen	301	2 117	142	34,60	8,21	31,27	12,85	7,11	109,77	4,14	63,96	2,97	45,80	72%
Mönchengladbach	260	170	1523	34,21	5,46	21,79	17,02	1,28	19,77	3,57	55,09	- 2,29	- 35,32	-64%
Mühldorf a. Inn	116	805	145	44,83	7,09	42,93	11,39	2,39	36,93	1,60	24,71	0,79	12,22	49%
Mülheim an der Ruhr	171	91	1872	49,05	8,92	27,69	25,22	0,78	12,06	2,35	36,26	- 1,57	- 24,20	-67%
München	1 488	311	4790	34,67	7,40	14,78	17,81	0,72	11,18	20,45	315,75	- 19,73	- 304,57	-96%
München	350	664	526	37,37	7,77	32,42	14,58	2,43	37,59	4,81	74,19	- 2,37	- 36,61	-49%
Münster	316	303	1043	39,08	7,08	30,88	17,11	2,06	31,88	4,35	67,13	- 2,28	- 35,25	-53%
Neckar-Odenwald-Kreis	144	1 126	128	53,70	6,49	50,63	13,96	4,40	67,88	1,98	30,51	2,42	37,37	122%
Neuburg-Schrobenhausen	98	740	132	45,95	5,70	42,67	13,04	2,05	31,68	1,34	20,73	0,71	10,95	53%
Neumarkt i.d. OPf.	135	1 344	101	51,36	7,17	49,68	11,53	3,47	53,60	1,86	28,69	1,61	24,91	87%
Neumünster	80	72	1115	48,23	5,32	29,63	23,84	0,30	4,61	1,10	16,95	- 0,80	- 12,35	-73%
Neunkirchen	131	250	525	48,79	6,83	37,76	21,27	0,58	9,02	1,80	27,83	- 1,22	- 18,81	-68%
Neustadt a.d. Aisch-Bad Windsheim	101	1 267	80	43,11	9,21	41,73	11,82	2,64	40,76	1,39	21,49	1,25	19,27	90%

County name	Population [tsd.]	Area [km²]	Population Density [inh. / km²]	RES supply [0-100] at SPAs (excl. SBAs)		RES supply [0-100] incl. SBAs (= 0)		RES use at indigenous SPAs [mil visits p.a.]	Income at indigenous SPAs [mil. EUR p.a.]	Demand at indigenous SBAs [mil. visits p.a.]	Expenses from indigenous SPAs [mil. EUR p.a.]	RES use balance [mil. visits p.a.]	RES-related benefit flow	
				MEAN	StD	MEAN	StD						[mil. EUR p.a.]	ratio
Neustadt a.d. Waldnaab	95	1 428	66	52,66	4,82	50,81	10,79	2,25	34,72	1,30	20,08	0,95	14,64	73%
Neustadt an der Weinstraße	53	117	455	64,06	7,43	54,80	23,55	0,91	14,01	0,73	11,31	0,17	2,70	24%
Neu-Ulm	176	516	341	46,96	6,08	41,54	16,06	1,82	28,05	2,42	37,30	- 0,60	- 9,25	-25%
Neuwied	183	627	292	57,91	6,03	52,02	18,41	4,82	74,49	2,52	38,85	2,31	35,63	92%
Nienburg (Weser)	122	1 401	87	37,58	8,81	35,02	12,73	3,96	61,20	1,67	25,81	2,29	35,39	137%
Nordfriesland	167	2 084	80	40,37	9,33	38,56	12,36	2,55	39,33	2,30	35,46	0,25	3,86	11%
Nordhausen	82	714	115	42,11	14,68	39,75	17,24	1,43	22,15	1,13	17,49	0,30	4,65	27%
Nordsachsen	197	2 029	97	34,28	9,54	32,40	12,13	4,46	68,83	2,71	41,89	1,74	26,94	64%
Nordwestmecklenburg	158	2 127	74	37,09	11,16	35,88	12,80	4,25	65,62	2,17	33,52	2,08	32,11	96%
Northeim	132	1 269	104	49,46	10,80	47,34	14,56	3,56	54,93	1,81	27,96	1,75	26,97	96%
Nürnberg	516	186	2765	36,22	6,54	19,79	18,67	0,35	5,35	7,08	109,38	- 6,74	- 104,03	-95%
Nürnberger Land	171	800	214	53,87	9,86	50,26	16,49	2,53	39,04	2,35	36,31	0,18	2,73	8%
Oberallgäu	156	1 528	102	60,40	9,15	58,88	13,08	2,70	41,76	2,15	33,16	0,56	8,59	26%
Oberbergischer Kreis	272	919	296	61,44	5,75	54,93	19,67	12,58	194,30	3,73	57,65	8,85	136,66	237%
Oberhausen	210	77	2718	39,04	5,81	16,80	19,70	0,52	7,95	2,88	44,46	- 2,36	- 36,51	-82%
Oberhavel	214	1 808	118	42,58	7,25	39,72	12,75	6,84	105,69	2,94	45,45	3,90	60,23	133%
Oberspreewald-Lausitz	108	1 223	89	43,71	7,89	40,89	13,16	3,43	52,97	1,49	23,00	1,94	29,97	130%
Odenwaldkreis	97	624	155	54,95	5,88	51,53	14,45	2,94	45,43	1,33	20,53	1,61	24,90	121%
Oder-Spree	179	2 257	79	40,46	8,66	38,16	12,59	6,96	107,41	2,46	38,04	4,49	69,38	182%
Offenbach	357	356	1001	42,67	6,36	33,21	18,59	1,50	23,09	4,90	75,65	- 3,40	- 52,56	-69%
Offenbach am Main	131	45	2916	40,55	5,21	26,67	19,70	0,17	2,57	1,80	27,77	- 1,63	- 25,20	-91%
Oldenburg	131	1 065	123	33,11	6,52	31,19	10,00	3,22	49,72	1,81	27,89	1,41	21,83	78%
Oldenburg (Oldb)	170	103	1645	39,28	8,05	19,55	20,45	0,22	3,37	2,33	35,98	- 2,11	- 32,61	-91%
Olpe	133	712	187	61,26	5,82	56,80	16,87	6,28	96,98	1,83	28,29	4,45	68,68	243%
Ortenaukreis	433	1 860	233	63,86	11,90	58,38	21,20	7,10	109,59	5,94	91,78	1,15	17,81	19%

County name	Population [tsd.]	Area [km²]	Population Density [inh. / km²]	RES supply [0-100] at SPAs (excl. SBAs)		RES supply [0-100] incl. SBAs (= 0)		RES use at indigenous SPAs [mil visits p.a.]	Income at indigenous SPAs [mil. EUR p.a.]	Demand at indigenous SBAs [mil. visits p.a.]	Expenses from indigenous SPAs [mil. EUR p.a.]	RES use balance [mil. visits p.a.]	RES-related benefit flow	
				MEAN	StD	MEAN	StD						[mil. EUR p.a.]	ratio
Osnabrück	359	2 122	169	35,35	7,18	33,12	11,05	7,60	117,27	4,94	76,27	2,66	41,00	54%
Osnabrück	164	120	1371	36,27	4,70	22,47	17,99	0,45	6,95	2,26	34,84	- 1,81	- 27,89	-80%
Ostalbkreis	314	1 511	208	55,57	7,43	51,73	15,80	5,22	80,62	4,32	66,68	0,90	13,94	21%
Ostallgäu	142	1 394	102	50,54	7,52	48,51	12,36	3,29	50,77	1,95	30,11	1,34	20,66	69%
Osterholz	115	653	176	46,08	5,10	41,14	15,04	1,78	27,44	1,58	24,32	0,20	3,12	13%
Ostholstein	201	1 393	145	37,78	12,43	35,74	14,80	3,03	46,79	2,77	42,75	0,26	4,05	9%
Ostprignitz-Ruppin	99	2 526	39	38,64	11,16	37,50	12,80	4,95	76,37	1,36	20,96	3,59	55,40	264%
Paderborn	308	1 247	247	44,58	9,54	40,76	15,45	5,25	81,03	4,24	65,42	1,01	15,61	24%
Passau	193	1 530	126	49,15	8,13	46,63	13,43	2,08	32,17	2,66	41,04	- 0,57	- 8,87	-22%
Passau	52	70	754	51,88	6,55	39,28	22,96	0,10	1,57	0,72	11,12	- 0,62	- 9,55	-86%
Peine	136	537	253	28,11	6,12	25,30	10,24	1,44	22,24	1,87	28,82	- 0,43	- 6,58	-23%
Pfaffenhofen a.d. Ilm	129	761	170	48,94	5,72	45,78	13,23	3,33	51,49	1,77	27,40	1,56	24,09	88%
Pforzheim	126	98	1286	61,66	7,75	46,09	27,61	0,71	10,96	1,73	26,74	- 1,02	- 15,77	-59%
Pinneberg	317	664	477	43,71	8,92	36,57	18,11	2,66	41,07	4,36	67,27	- 1,70	- 26,20	-39%
Pirmasens	40	61	655	59,11	5,65	45,07	25,64	0,22	3,46	0,55	8,52	- 0,33	- 5,07	-59%
Plön	129	1 084	119	46,46	9,95	44,44	13,57	3,37	52,02	1,78	27,44	1,59	24,58	90%
Potsdam	182	188	967	49,87	6,69	40,37	20,48	0,95	14,70	2,50	38,64	- 1,55	- 23,93	-62%
Potsdam-Mittelmark	218	2 592	84	42,08	7,49	39,98	11,72	11,34	175,11	2,99	46,24	8,35	128,87	279%
Prignitz	76	2 139	36	32,28	8,13	31,53	9,39	1,06	16,34	1,05	16,14	0,01	0,19	1%
Rastatt	232	738	314	64,40	9,43	57,44	21,88	4,10	63,34	3,19	49,24	0,91	14,10	29%
Ravensburg	286	1 632	175	54,68	5,72	52,36	12,36	3,95	61,00	3,93	60,66	0,02	0,34	1%
Recklinghausen	614	761	806	40,49	7,08	31,45	17,97	6,93	107,06	8,43	130,18	- 1,50	- 23,12	-18%
Regen	77	975	79	59,00	4,45	57,29	10,82	1,38	21,37	1,06	16,40	0,32	4,97	30%
Regensburg	152	81	1883	39,83	7,69	22,01	20,61	0,16	2,53	2,09	32,31	- 1,93	- 29,77	-92%
Regensburg	194	1 392	140	45,01	10,81	42,93	14,17	3,13	48,37	2,67	41,22	0,46	7,15	17%

Title: Modelling flows of RES from landscapes to people: Insights from Germany

County name	Population [tsd.]	Area [km²]	Population Density [inh. / km²]	RES supply [0-100] at SPAs (excl. SBAs)		RES supply [0-100] incl. SBAs (= 0)		RES use at indigenous SPAs [mil visits p.a.]	Income at indigenous SPAs [mil. EUR p.a.]	Demand at indigenous SBAs [mil. visits p.a.]	Expenses from indigenous SPAs [mil. EUR p.a.]	RES use balance [mil. visits p.a.]	RES-related benefit flow	
				MEAN	StD	MEAN	StD						[mil. EUR p.a.]	ratio
Region Hannover	1 155	2 297	503	35,74	8,86	30,75	14,87	7,16	110,54	15,88	245,12	- 8,72	- 134,58	-55%
Regionalverband Saarbrücken	328	411	797	48,39	6,82	36,07	21,88	0,81	12,45	4,50	69,48	- 3,69	- 57,04	-82%
Remscheid	112	75	1496	52,80	9,67	33,41	26,59	0,82	12,65	1,53	23,66	- 0,71	- 11,01	-47%
Rems-Murr-Kreis	427	858	498	64,11	7,21	57,12	21,11	5,89	91,00	5,87	90,66	0,02	0,34	0%
Rendsburg-Eckernförde	275	2 190	125	43,62	9,27	41,33	13,27	7,62	117,73	3,78	58,30	3,85	59,43	102%
Reutlingen	287	1 092	263	59,98	7,88	55,20	17,92	5,35	82,54	3,95	61,00	1,40	21,55	35%
Rhein-Erft-Kreis	470	705	666	33,82	9,80	25,63	16,81	5,33	82,24	6,45	99,64	- 1,13	- 17,39	-17%
Rheingau-Taunus-Kreis	187	811	231	59,27	8,66	55,95	16,01	5,25	81,07	2,58	39,77	2,67	41,30	104%
Rhein-Hunsrück-Kreis	103	991	104	54,98	7,58	53,35	11,94	4,22	65,09	1,42	21,94	2,79	43,15	197%
Rheinisch-Bergischer Kreis	283	437	648	60,05	6,70	49,91	23,31	6,42	99,08	3,89	60,10	2,52	38,98	65%
Rhein-Kreis Neuss	452	576	784	31,88	8,88	23,79	15,85	4,66	71,88	6,21	95,90	- 1,56	- 24,02	-25%
Rhein-Lahn-Kreis	123	782	157	56,28	8,33	53,82	14,09	4,23	65,35	1,68	26,01	2,55	39,34	151%
Rhein-Neckar-Kreis	548	1 062	516	55,02	10,41	47,88	20,88	6,56	101,23	7,53	116,32	- 0,98	- 15,09	-13%
Rhein-Pfalz-Kreis	155	305	507	53,31	7,40	45,43	20,11	1,69	26,12	2,13	32,83	- 0,44	- 6,72	-20%
Rhein-Sieg-Kreis	600	1 153	521	52,35	11,29	44,80	21,16	11,04	170,42	8,25	127,38	2,79	43,04	34%
Rhön-Grabfeld	80	1 022	78	48,36	11,20	46,46	14,45	1,70	26,30	1,09	16,87	0,61	9,43	56%
Rosenheim	64	37	1709	55,04	6,34	35,22	26,90	0,12	1,89	0,87	13,49	- 0,75	- 11,60	-86%
Rosenheim	262	1 439	182	55,75	7,34	52,54	14,81	5,36	82,77	3,60	55,53	1,76	27,24	49%
Rostock	217	3 431	63	35,00	10,25	34,04	11,61	3,72	57,43	2,98	46,06	0,74	11,37	25%
Rostock	209	181	1153	39,01	6,59	29,31	17,81	0,18	2,82	2,87	44,36	- 2,69	- 41,53	-94%
Rotenburg (Wümme)	164	2 075	79	45,50	4,81	42,71	11,86	7,41	114,38	2,26	34,90	5,15	79,48	228%
Roth	127	895	142	46,92	6,53	44,49	12,20	2,37	36,61	1,75	26,98	0,62	9,63	36%
Rottal-Inn	122	1 281	95	45,50	4,99	43,97	9,56	2,07	31,91	1,67	25,84	0,39	6,07	23%
Rottweil	140	769	182	57,98	6,05	52,93	17,34	3,23	49,86	1,93	29,74	1,30	20,12	68%
Saale-Holzland-Kreis	83	815	102	45,50	8,47	43,85	11,88	2,99	46,23	1,14	17,57	1,86	28,66	163%

County name	Population [tsd.]	Area [km²]	Population Density [inh. / km²]	RES supply [0-100] at SPAs (excl. SBAs)		RES supply [0-100] incl. SBAs (= 0)		RES use at indigenous SPAs [mil visits p.a.]	Income at indigenous SPAs [mil. EUR p.a.]	Demand at indigenous SBAs [mil. visits p.a.]	Expenses from indigenous SPAs [mil. EUR p.a.]	RES use balance [mil. visits p.a.]	RES-related benefit flow	
				MEAN	StD	MEAN	StD						[mil. EUR p.a.]	ratio
Saalekreis	183	1 434	128	29,71	11,78	27,42	13,81	3,31	51,09	2,52	38,92	0,79	12,17	31%
Saale-Orla-Kreis	80	1 151	69	49,43	7,93	48,02	11,35	3,25	50,12	1,09	16,90	2,15	33,22	197%
Saalfeld-Rudolstadt	102	1 009	101	52,95	6,88	50,76	12,52	2,02	31,21	1,40	21,67	0,62	9,54	44%
Saarlouis	194	459	422	49,25	6,79	40,01	20,18	0,94	14,48	2,66	41,10	- 1,72	- 26,62	-65%
Saarpfalz-Kreis	142	418	339	50,65	7,34	42,85	19,49	1,05	16,17	1,95	30,05	- 0,90	- 13,88	-46%
Sächsische Schweiz-Osterzgebirge	245	1 654	148	47,25	9,66	43,17	16,17	4,45	68,66	3,36	51,92	1,08	16,74	32%
Salzgitter	104	224	463	31,80	10,76	25,90	15,72	0,58	8,96	1,43	22,04	- 0,85	- 13,08	-59%
Salzlandkreis	187	1 428	131	25,50	9,00	23,49	11,03	2,54	39,18	2,58	39,77	- 0,04	- 0,59	-1%
Schaumburg	158	676	234	39,10	8,88	35,18	14,46	2,75	42,46	2,18	33,61	0,57	8,86	26%
Schleswig-Flensburg	203	2 071	98	38,41	8,96	36,90	11,52	3,47	53,61	2,78	42,99	0,69	10,62	25%
Schmalkalden-Meiningen	124	1 251	99	57,04	6,63	53,83	14,64	2,38	36,82	1,71	26,36	0,68	10,46	40%
Schwabach	41	41	1006	49,97	4,28	35,25	23,06	0,11	1,63	0,56	8,71	- 0,46	- 7,08	-81%
Schwäbisch Hall	198	1 484	133	51,19	10,56	48,86	14,85	4,73	73,02	2,72	41,98	2,01	31,04	74%
Schwalm-Eder-Kreis	180	1 539	117	47,11	10,28	44,56	14,61	3,25	50,19	2,47	38,16	0,78	12,03	32%
Schwandorf	148	1 458	102	51,50	4,87	49,06	11,93	2,63	40,60	2,04	31,50	0,59	9,10	29%
Schwarzwald-Baar-Kreis	213	1 025	208	53,85	6,42	50,15	14,98	2,95	45,56	2,93	45,16	0,03	0,40	1%
Schweinfurt	53	36	1494	41,60	10,41	24,85	21,93	0,06	0,88	0,73	11,31	- 0,68	- 10,44	-92%
Schweinfurt	116	841	137	41,68	10,10	39,58	13,42	1,57	24,20	1,59	24,54	- 0,02	- 0,34	-1%
Schwerin	96	131	733	44,89	5,85	36,62	18,18	0,23	3,56	1,31	20,28	- 1,08	- 16,72	-82%
Segeberg	278	1 344	207	41,85	9,50	37,95	15,16	6,17	95,20	3,82	58,98	2,35	36,21	61%
Siegen-Wittgenstein	275	1 133	243	61,50	6,05	55,00	19,76	5,69	87,79	3,79	58,45	1,90	29,34	50%
Sigmaringen	131	1 204	109	53,10	5,61	50,30	13,06	3,04	46,89	1,80	27,78	1,24	19,11	69%
Soest	301	1 329	227	35,26	10,52	32,10	14,22	7,36	113,61	4,14	63,87	3,22	49,74	78%

Title: Modelling flows of RES from landscapes to people: Insights from Germany

County name	Population [tsd.]	Area [km^2]	Population Density [inh. / km^2]	RES supply [0-100] at SPAs (excl. SBAs)		RES supply [0-100] incl. SBAs (= 0)		RES use at indigenous SPAs [mil visits p.a.]	Income at indigenous SPAs [mil. EUR p.a.]	Demand at indigenous SBAs [mil. visits p.a.]	Expenses from indigenous SPAs [mil. EUR p.a.]	RES use balance [mil. visits p.a.]	RES-related benefit flow	
				MEAN	StD	MEAN	StD						[mil. EUR p.a.]	ratio
Solingen	159	90	1778	55,37	12,75	31,25	29,08	1,20	18,55	2,19	33,78	- 0,99	- 15,22	-45%
Sömmerda	69	807	86	27,86	10,92	26,66	12,09	1,12	17,29	0,95	14,66	0,17	2,63	18%
Sonneberg	57	461	124	53,73	4,84	50,17	14,17	0,76	11,79	0,78	12,10	- 0,02	- 0,31	-3%
Speyer	51	43	1188	58,40	5,06	38,65	27,93	0,25	3,91	0,70	10,77	- 0,44	- 6,86	-64%
Spree-Neiße	113	1 657	68	41,78	7,63	38,33	13,61	1,96	30,33	1,55	23,98	0,41	6,35	27%
St. Wendel	86	476	182	51,86	5,34	46,97	15,99	1,36	20,92	1,19	18,34	0,17	2,58	14%
Stade	205	1 267	162	44,83	5,40	41,08	13,46	3,68	56,89	2,82	43,57	0,86	13,32	31%
Städteregion Aachen	557	707	787	48,24	11,07	38,32	21,85	3,94	60,79	7,65	118,10	- 3,71	- 57,30	-49%
Starnberg	137	488	280	49,01	8,56	44,37	16,49	1,98	30,52	1,88	28,98	0,10	1,54	5%
Steinburg	131	1 056	124	43,03	8,70	40,03	13,81	3,96	61,10	1,80	27,73	2,16	33,37	120%
Steinfurt	448	1 796	250	34,51	6,47	31,45	11,59	7,99	123,31	6,16	95,09	1,83	28,21	30%
Stendal	110	2 424	46	34,82	10,26	33,74	11,77	1,41	21,74	1,52	23,44	- 0,11	- 1,71	-7%
Stormarn	245	766	320	36,56	10,46	32,61	15,05	3,28	50,67	3,37	51,98	- 0,08	- 1,30	-3%
Straubing	48	68	704	30,30	9,54	24,09	14,90	0,08	1,30	0,65	10,10	- 0,57	- 8,80	-87%
Straubing-Bogen	102	1 202	85	45,71	14,75	44,17	16,67	2,25	34,78	1,40	21,59	0,85	13,19	61%
Stuttgart	630	207	3040	50,95	9,81	30,43	26,11	0,84	12,90	8,66	133,73	- 7,83	- 120,83	-90%
Südliche Weinstraße	111	640	173	64,26	7,89	60,76	16,48	4,64	71,69	1,52	23,50	3,12	48,18	205%
Südwestpfalz	95	954	100	58,45	6,46	55,63	14,01	3,62	55,82	1,30	20,14	2,31	35,68	177%
Suhl	36	142	257	58,28	6,66	51,48	19,72	0,35	5,45	0,50	7,72	- 0,15	- 2,27	-29%
Teltow-Fläming	172	2 104	82	39,12	9,85	37,12	12,89	7,63	117,75	2,36	36,40	5,27	81,35	224%
Tirschenreuth	72	1 084	66	50,79	5,47	49,02	10,77	1,31	20,22	0,99	15,21	0,32	5,01	33%
Traunstein	177	1 534	116	54,41	9,30	52,30	13,90	3,50	54,07	2,44	37,66	1,06	16,41	44%
Trier	111	117	945	62,78	7,59	48,81	26,95	0,35	5,46	1,52	23,48	- 1,17	- 18,03	-77%
Trier-Saarburg	151	1 102	137	59,36	9,22	56,62	15,37	3,48	53,78	2,07	31,94	1,41	21,84	68%
Tübingen	228	519	440	61,64	6,28	54,61	20,47	3,08	47,55	3,14	48,47	- 0,06	- 0,93	-2%
Tuttlingen	142	734	193	54,27	5,32	50,10	15,32	1,85	28,53	1,95	30,06	- 0,10	- 1,53	-5%

Title: Modelling flows of RES from landscapes to people: Insights from Germany

County name	Population [tsd.]	Area [km²]	Population Density [inh. / km²]	RES supply [0-100] at SPAs (excl. SBAs)		RES supply [0-100] incl. SBAs (= 0)		RES use at indigenous SPAs [mil visits p.a.]	Income at indigenous SPAs [mil. EUR p.a.]	Demand at indigenous SBAs [mil. visits p.a.]	Expenses from indigenous SPAs [mil. EUR p.a.]	RES use balance [mil. visits p.a.]	RES-related benefit flow	
				MEAN	StD	MEAN	StD						[mil. EUR p.a.]	ratio
Uckermark	118	3 077	38	36,68	12,84	35,46	14,24	3,85	59,48	1,62	25,09	2,23	34,39	137%
Uelzen	93	1 463	63	36,96	8,26	35,87	10,26	2,06	31,86	1,27	19,64	0,79	12,23	62%
Ulm	126	119	1065	50,28	9,81	38,27	23,08	0,45	6,91	1,74	26,82	- 1,29	- 19,91	-74%
Unna	394	543	725	35,96	8,63	28,35	16,57	4,82	74,37	5,41	83,51	- 0,59	- 9,15	-11%
Unstrut-Hainich-Kreis	102	980	104	37,20	13,10	35,44	15,03	1,34	20,70	1,40	21,58	- 0,06	- 0,88	-4%
Unterallgäu	146	1 230	119	48,55	5,30	45,85	12,26	4,16	64,25	2,01	31,01	2,15	33,24	107%
Vechta	144	814	176	32,45	8,33	29,58	12,17	2,33	35,95	1,97	30,49	0,35	5,46	18%
Verden	138	789	174	40,43	7,23	36,96	13,26	2,87	44,34	1,89	29,19	0,98	15,15	52%
Viersen	299	563	530	33,22	6,87	27,17	14,25	4,46	68,80	4,10	63,34	0,35	5,46	9%
Vogelsbergkreis	106	1 459	72	58,07	8,45	56,21	13,18	4,92	75,96	1,45	22,38	3,47	53,57	239%
Vogtlandkreis	224	1 412	159	49,18	6,63	44,53	15,71	3,45	53,31	3,08	47,50	0,38	5,80	12%
Vorpommern-Greifswald	236	3 946	60	35,05	11,21	33,86	12,72	2,61	40,37	3,24	50,02	- 0,63	- 9,66	-19%
Vorpommern-Rügen	225	3 216	70	36,74	12,88	35,57	14,22	2,83	43,75	3,10	47,82	- 0,26	- 4,06	-9%
Vulkaneifel	60	912	66	58,30	6,46	56,33	12,30	2,96	45,73	0,83	12,83	2,13	32,90	256%
Waldeck-Frankenberg	157	1 849	85	50,57	8,56	48,52	13,02	3,85	59,45	2,15	33,21	1,70	26,24	79%
Waldshut	171	1 131	151	60,92	6,28	57,57	15,17	2,98	45,99	2,35	36,33	0,63	9,66	27%
Warendorf	277	1 319	210	30,19	5,37	28,07	9,29	6,82	105,30	3,81	58,86	3,01	46,44	79%
Wartburgkreis	118	1 267	93	53,55	10,45	50,90	15,45	2,54	39,27	1,62	25,03	0,92	14,24	57%
Weiden i.d. OPf.	43	71	603	52,34	3,75	41,14	21,72	0,11	1,65	0,58	9,02	- 0,48	- 7,37	-82%
Weilheim-Schongau	136	966	141	53,19	5,12	50,42	12,84	3,00	46,36	1,87	28,88	1,13	17,48	61%
Weimar	65	84	771	48,74	10,15	40,16	20,72	0,20	3,08	0,89	13,81	- 0,70	- 10,73	-78%
Weimarer Land	82	804	102	39,72	11,65	38,26	13,65	1,88	29,03	1,13	17,46	0,75	11,57	66%
Weißenburg-Gunzenhausen	95	971	98	47,44	6,19	45,15	11,83	2,05	31,61	1,31	20,18	0,74	11,43	57%
Werra-Meißner-Kreis	100	1 025	98	55,88	6,54	53,23	13,48	2,41	37,17	1,37	21,23	1,03	15,94	75%

Title: Modelling flows of RES from landscapes to people: Insights from Germany

County name	Population [tsd.]	Area [km²]	Population Density [inh. / km²]	RES supply [0-100] at SPAs (excl. SBAs)		RES supply [0-100] incl. SBAs (= 0)		RES use at indigenous SPAs [mil visits p.a.]	Income at indigenous SPAs [mil. EUR p.a.]	Demand at indigenous SBAs [mil. visits p.a.]	Expenses from indigenous SPAs [mil. EUR p.a.]	RES use balance [mil. visits p.a.]	RES-related benefit flow	
				MEAN	StD	MEAN	StD						[mil. EUR p.a.]	ratio
Wesel	460	1 043	441	43,49	7,47	37,46	16,55	9,99	154,28	6,32	97,62	3,67	56,66	58%
Wesermarsch	89	825	107	42,25	5,71	39,86	11,24	2,33	35,97	1,22	18,78	1,11	17,19	92%
Westerwaldkreis	203	989	205	56,96	5,43	52,33	16,42	5,23	80,76	2,79	43,03	2,44	37,73	88%
Wetteraukreis	310	1 101	282	49,83	14,08	45,75	19,19	4,84	74,66	4,26	65,85	0,57	8,81	13%
Wiesbaden	279	204	1367	48,87	12,86	36,39	24,03	0,98	15,13	3,83	59,11	- 2,85	- 43,98	-74%
Wilhelmshaven	75	107	702	44,20	5,22	29,48	21,26	0,20	3,10	1,03	15,95	- 0,83	- 12,85	-81%
Wittenberg	124	1 932	64	38,23	8,24	36,56	11,22	3,82	58,93	1,71	26,35	2,11	32,59	124%
Wittmund	57	657	87	42,95	5,82	41,17	10,28	1,28	19,70	0,79	12,17	0,49	7,53	62%
Wolfenbüttel	119	724	165	31,72	10,11	29,93	12,25	1,89	29,20	1,64	25,32	0,25	3,88	15%
Wolfsburg	124	205	605	44,88	5,98	35,29	19,15	0,55	8,56	1,70	26,27	- 1,15	- 17,72	-67%
Worms	83	109	768	57,25	8,61	45,32	24,49	0,74	11,50	1,15	17,71	- 0,40	- 6,21	-35%
Wunsiedel i. Fichtelgebirge	72	606	119	51,67	4,32	48,77	12,63	0,96	14,75	0,99	15,27	- 0,03	- 0,52	-3%
Wuppertal	355	168	2108	51,65	10,44	31,61	26,46	1,91	29,43	4,88	75,32	- 2,97	- 45,89	-61%
Würzburg	163	968	168	39,62	13,42	37,17	16,12	2,17	33,56	2,24	34,52	- 0,06	- 0,96	-3%
Würzburg	127	88	1449	42,62	8,19	27,55	21,41	0,23	3,52	1,74	26,94	- 1,52	- 23,42	-87%
Zollernalbkreis	190	918	207	58,65	6,19	53,05	18,21	3,57	55,05	2,61	40,28	0,96	14,77	37%
Zweibrücken	34	71	481	51,11	4,79	39,75	21,67	0,17	2,58	0,47	7,21	- 0,30	- 4,63	-64%
Zwickau	312	950	329	36,88	8,08	30,09	16,05	3,08	47,62	4,29	66,20	- 1,20	- 18,58	-28%
Germany	83 155	357 580	233	45,87	12,44	42,29	17,15	1 142,66	17 642,66	1 142,66	17 642,66	0,00	0,01	0%

Figure S4: Modelled RES Flow in Germany considering maximum 180-minute travel time

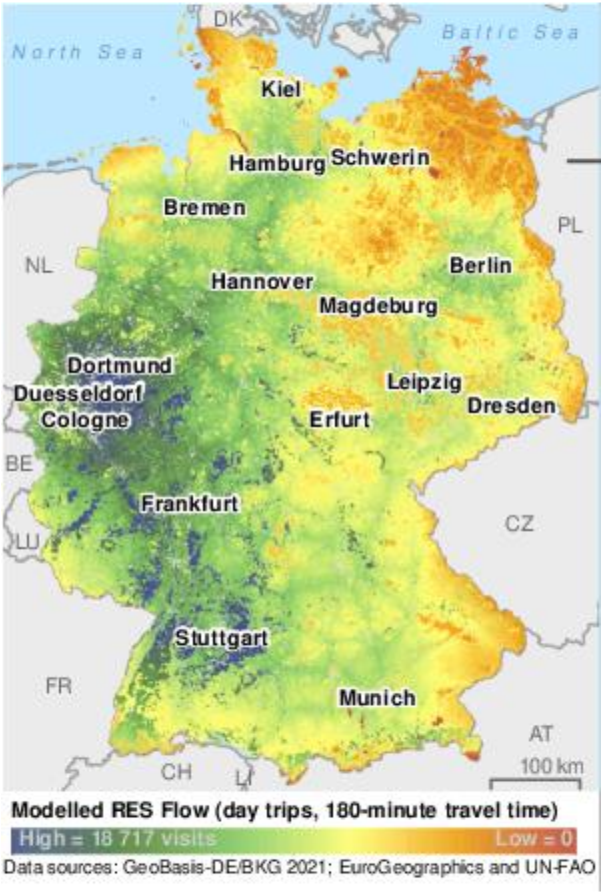

Supplement: Supplementary file 1 — Supplementary file1 (PDF 1218 KB) [file 13280_2024_2081_MOESM1_ESM.pdf]
